# PACAP suppresses dry eye signs by stimulating tear secretion

**DOI:** 10.1038/ncomms12034

**Published:** 2016-06-27

**Authors:** Tomoya Nakamachi, Hirokazu Ohtaki, Tamotsu Seki, Sachiko Yofu, Nobuyuki Kagami, Hitoshi Hashimoto, Norihito Shintani, Akemichi Baba, Laszlo Mark, Ingela Lanekoff, Peter Kiss, Jozsef Farkas, Dora Reglodi, Seiji Shioda

**Affiliations:** 1 Laboratory of Regulatory Biology, Graduate School of Science and Engineering, University of Toyama, 3190-Gofuku, Toyama-shi, Toyama 930-8555, Japan; 2 Department of Anatomy, Showa University School of Medicine, Shinagawa-Ku, Tokyo 142-8555, Japan; 3 Laboratory of Molecular Neuropharmacology, Graduate School of Pharmaceutical Sciences, Osaka University, 1-6 Yamadaoka, Suita, Osaka 565-0871, Japan; 4 iPS Cell-based Research Project on Brain Neuropharmacology and Toxicology, Graduate School of Pharmaceutical Sciences, Osaka University, 1-6 Yamadaoka, Suita, Osaka 565-0871, Japan; 5 Molecular Research Center for Children's Mental Development, United Graduate School of Child Development, Osaka University, Kanazawa University, Hamamatsu University School of Medicine, Chiba University and University of Fukui, 2-2 Yamadaoka, Suita, Osaka 565-0871, Japan; 6 Hyogo University of Health Sciences, 1-3-6 Minatojima, Chuo-ku, Kobe, Hyogo 650-8530, Japan; 7 Department of Analytical Biochemistry, Institute of Biochemistry and Medical Chemistry, Medical School, University of Pécs, Szigeti u 12, Pécs 7624, Hungary; 8 Imaging Center for Life and Material Sciences, University of Pécs, Szigeti u 12, Pécs 7624, Hungary; 9 János Szentágothai Research Center, University of Pécs, Szigeti u 12, Pécs 7624, Hungary; 10 PTE-MTA Human Reproduction Research Group, Szigeti u 12, Pécs 7624, Hungary; 11 Department of Chemistry-BMC, Uppsala University, PO Box 599, Uppsala 751 24, Sweden; 12 Department of Anatomy, MTA-PTE PACAP Lendulet Research Group, Centre for Neuroscience, University of Pécs, Szigeti u 12, Pécs 7624, Hungary; 13 Innovative Drug Discovery, Global Research Center for Innovative Life Science, Hoshi University, 4-41 Ebara 2-chome, Shinagawa-ku, Tokyo 142-8501, Japan

## Abstract

Dry eye syndrome is caused by a reduction in the volume or quality of tears. Here, we show that pituitary adenylate cyclase-activating polypeptide (PACAP)-null mice develop dry eye-like symptoms such as corneal keratinization and tear reduction. PACAP immunoreactivity is co-localized with a neuronal marker, and PACAP receptor (PAC1-R) immunoreactivity is observed in mouse infraorbital lacrimal gland acinar cells. PACAP eye drops stimulate tear secretion and increase cAMP and phosphorylated (p)-protein kinase A levels in the infraorbital lacrimal glands that could be inhibited by pre-treatment with a PAC1-R antagonist or an adenylate cyclase inhibitor. Moreover, these eye drops suppress corneal keratinization in PACAP-null mice. PACAP eye drops increase aquaporin 5 (AQP5) levels in the membrane and pAQP5 levels in the infraorbital lacrimal glands. AQP5 siRNA treatment of the infraorbital lacrimal gland attenuates PACAP-induced tear secretion. Based on these results, PACAP might be clinically useful to treat dry eye disorder.

## Abstract (web summary)

Dry eye disease is a complex condition with limited treatments. Here the authors show that mice lacking a multi-functional peptide PACAP develop dry eye-like signs that can be topically treated with PACAP peptide that stimulates tearing in mice, suggesting a possible therapy in humans with dry eyes.

## Graphical abstract

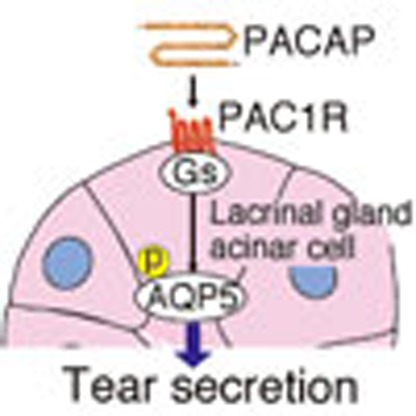

Dry eye syndrome, also known as keratoconjunctivitis sicca, is a common eye disease caused by a reduction in the volume or quality of tears. Tear components are secreted from the main lacrimal gland, accessory lacrimal gland (Krause and Wolfring glands), meibomian gland, and the corneal and conjunctival epithelia in humans. A thin layer of tear film containing water, lipid electrolytes and ∼10 mg ml^−1^ protein comprising different tear proteins, covers the ocular surface, thereby maintaining and protecting the eye. The major categories of dry eye are the aqueous tear-deficient type, in which the lacrimal glands fail to produce enough of the watery component of tears to maintain a tear film, and the evaporative type, in which impaired lipid secretion from the meibomian glands destabilizes the tear film^1^. Dry eye syndrome correlates with old age and affects females to a larger degree^2^. The number of patients diagnosed with the condition has increased in recent years, which could be due to the popularity of video display use (computer vision syndrome) or the wearing of contact lenses^3^,^4^. The orthodox strategy for the treatment of dry eye syndrome is symptomatic therapy, such as tear replacement using artificial tears. Although artificial tears provide temporary symptomatic relief, they do not address the underlying pathophysiology of dry eye syndrome, and the outcome is not always satisfactory^5^.

Pituitary adenylate cyclase-activating polypeptide (PACAP; encoded by the gene *Adcyap1*), which exists in 27- or 38-amino-acid isoforms, was originally discovered in extracts of ovine hypothalamus^6^,^7^. The amino-acid sequence of PACAP—a member of the vasoactive intestinal polypeptide (VIP)/secretin/growth hormone-releasing hormone family of peptides—shows a 68% sequence homology with VIP. PACAP and VIP share three different receptors: the VPAC1 and VPAC2 receptors (VPAC1-R and VPAC2-R; gene names: *Vipr1* and *Vipr2*) and the PAC1-receptor (PAC1-R; encoded by the gene: *Adcyap1r1*) with different splice variants^8^,^9^. The affinity of PAC1-R for PACAP is more than 1,000 times higher than its affinity for VIP, indicating that PAC1-R is a relatively selective receptor for PACAP (ref. 10). The amino-acid sequences of PACAP27 and PACAP38 are highly conserved among mammals, and those of mouse and human are identical. PACAP is distributed mainly in the central nervous system, but it is also detected in the testis, adrenal gland, digestive tract and other peripheral organs^10^. The amino-acid sequence of PAC1-R is conserved with a 96.6% homology between mouse and human. *Adcyap1r1* mRNA is widely detected in the central nervous system and peripheral organs, including the adrenal gland, testis, anterior pituitary gland, pancreas and placenta^10^.

PACAP is a multi-functional peptide that can act as a neurotrophic factor, neuroprotectant, neurotransmitter, immunomodulator and vasodilator^10^,^11^,^12^,^13^. During the past 10 years, PACAP-null (*Adcyap1*^*−/−*^) mice have been generated by several laboratories and their phenotypes have been analysed^14^,^15^,^16^. Recently, we observed that corneal keratinization, associated with a decreased tear volume, frequently occurs in *Adcyap1*^*−/−*^ mice. It has been reported that PACAP immunopositive nerve fibres were observed in cat lacrimal gland^17^, but PACAP and PACAP receptor expression and function in lacrimal glands has not been well studied to date. To address this interesting finding, we investigate the effects and underlying mechanism of action of PACAP on lacrimal gland tear secretion in PACAP-null mice, as well as in mice treated with topical eye drops.

## Results

### Dry eye-like signs in the *Adcyap1* ^*−/−*^ mouse

**Figure 1:**
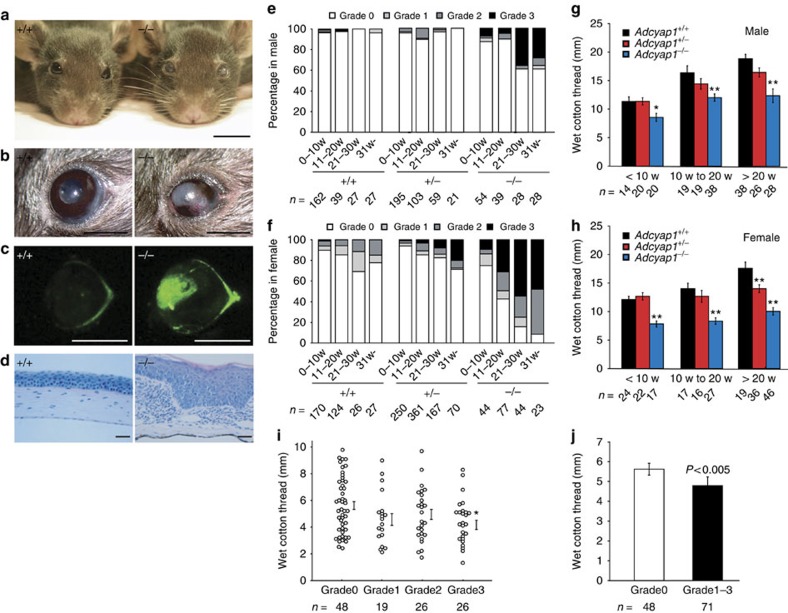
Corneal keratinization and reduction of tear volume in *Adcyap1*^*−/−*^ mice. (**a**–**d**) Low- and high-power images of the corneal surface showing pathological changes. Scale bars, 10 mm in **a**, 2 mm in **b** and **c**, and 50 μm in **d**. (**e**,**f**) Scoring of corneal keratinization in wild-type, *Adcyap1*^*+/−*^ and *Adcyap1*^*−/−*^ mice as described in Supplementary Fig. 1. The classification was evaluated on both sides, and the highest grade was used for the grading of male (**e**) and female (**f**) mice at different ages. (**g**,**h**) Tear volume measured with the cotton thread method in wild-type, *Adcyap1*^*+/−*^ and *Adcyap1*^*−/−*^ mice at different ages. The total wet cotton thread length from both eyes is shown for male (**g**) and female (**h**) mice. *P<0.05, **P<0.01 versus wild-type mice. (**i**,**j**) Relationship between the wet cotton thread length and the corneal grade in female *Adcyap1*^*−/−*^ mice. The dot plot graph shows the wet thread length for each eye of each grade expressed in terms of the mean±s.e. value (**i**). *P<0.05 versus Grade 0. The bar graph (**j**) shows the wet thread length of Grade 0 and Grades 1–3, based on the data in Fig. 1i.

During the routine housing of *Adcyap1*^*−/−*^ mice in our animal facility, we unexpectedly discovered that some mice exhibited cloudiness of the cornea (Fig. 1a). The ocular surface appeared white and sandy, and blood vessels could be seen in the cornea (Fig. 1b). Based on fluorescein staining, which is commonly used to visualize corneal injury, strong fluorescence was observed in the central part of the cornea in these mice (Fig. 1c). On examination of this pathology, we discovered that the corneal epithelial cells were hypertrophied and the surface was keratinized (Fig. 1d). To quantify the degree of corneal keratinization, corneas were classified into four grades with the aid of a dissecting microscope (from Grade 0, denoting normal, to Grade 3 signifying hypertrophy of the surface and keratinization, as shown in Supplementary Fig. 1). Wild-type and *Adcyap*^*+/−*^ male mice over the age of 20 weeks had normal corneas, whereas about 40% of *Adcyap1*^*−/−*^ male mice over 30 weeks of age had Grade 3 corneas (Fig. 1e). In female mice, all groups showed a higher frequency of keratinization than that observed in male mice (Fig. 1f). In female *Adcyap1*^*−/−*^ mice, the percentage of animals showing corneal keratinization was <20% in animals younger than 10 weeks of age, but increased with age to 90% of animals over 30 weeks of age (Fig. 1f). These data indicate that corneal keratinization frequently occurs in older *Adcyap1*^*−/−*^ mice, and particularly in female animals.

Because keratinization is a common feature of dry eye disorder, we postulated that the corneal keratinization was caused by a reduction in tear fluid or quality. To test this, the tear secretion level in *Adcyap1*^*−/−*^ mice was measured using the cotton thread method. As expected, tear secretion levels of male and female *Adcyap1*^*−/−*^ mice were reduced compared with those of wild-type mice aged 10 weeks or younger (Fig. 1g,h). The tear volume in eyes with corneal keratinization was significantly reduced compared with that of Grade 0 eyes (Fig. 1i,j), while the tear volume and the corneal grade were weakly though significantly inversely correlated (*r*=−0.242, *P*=0.007, two-tailed Spearman's correlation test). On histological examination, the infraorbital lacrimal gland, conjunctiva and corneal neural network of *Adcyap1*^*−/−*^ mice were found to be morphologically normal (Supplementary Fig. 2a–c). Taken together, these observations suggest that *Adcyap1*^*−/−*^ mice exhibit a dry eye-like phenotype with a reduction in tear volume and corneal damage, despite the structure of the infraorbital lacrimal gland, conjunctiva and neural network of the cornea remaining normal.

### Distribution and function of PACAP in the lacrimal gland

**Figure 2:**
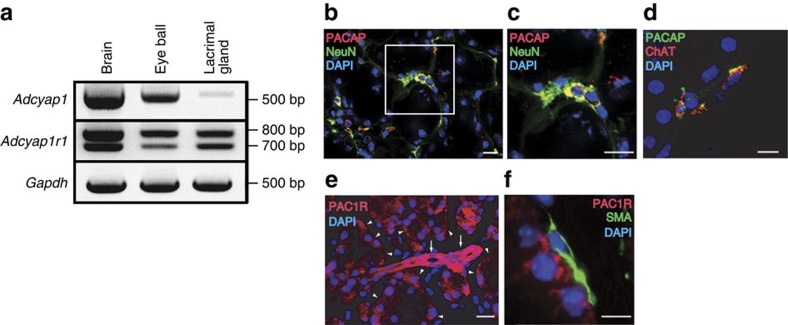
PACAP and PAC1-R expression in wild-type male mouse infraorbital lacrimal gland. (**a**) Reverse transcriptase PCR analysis to detect *Adcyap1* and *Adcyap1r1* mRNA in the infraorbital lacrimal gland. The eye ball was used as a positive control. (**b**–**d**) PACAP immunoreactivity in the mouse infraorbital lacrimal gland with NeuN immunoreactivity as a neuronal marker (**b**,**c**), and ChAT immunoreactivity as a marker of parasympathetic neurons (**d**). The white square in **b** is expanded in **c**. Scale bar, 20 μm in **b** and **c** and 10 μm in **d**. (**e**,**f**) PAC1-R immunoreactivity in the infraorbital lacrimal gland identified with single immunostaining in the acinus (arrow head) and in the duct (arrow) (**e**), and with smooth muscle actin (SMA) immunoreactivity as a myoepithelial cell marker (**f**). Scale bar, 20 μm in **e** and 5 μm in **f**. DAPI was used as a nuclear marker (**e**,**f**).

Based on the above data, we hypothesized that the PACAP/PAC1-R system was associated with altered tear secretion by the lacrimal gland, the major source of tear secretion. To test this, we first examined PACAP/PAC1-R expression and distribution in the mouse infraorbital lacrimal gland. Using the RT-PCR method, *Adcyap1* and *Adcyap1r1* mRNAs were detected in gland extracts, producing a signal with the same band size as that obtained from an eye ball sample that was used as a positive control (Fig. 2a). PACAP immunoreactivity was observed around acinar cells, and co-localized with immunoreactivity for the neuronal marker NeuN (Fig. 2b,c), and the parasympathetic neuronal marker choline acetyltransferase (ChAT) (Fig. 2d). The PACAP antibody recognized PACAP38 but not VIP (Supplementary Fig. 3a). PAC1-R immunoreactivity was observed on the basal side of acinar cells and ducts, but did not co-localize with smooth muscle actin as a myoepithelial cell marker (Fig. 2e,f). The PACAP and PAC1-R immunoreactivities were abolished by pre-absorption of the antibody with antigen (Supplementary Fig. 3b,c). PACAP38 was detected in wild-type, but not *Adcyap1*^*−/−*^ mouse tears by matrix-assisted laser desorption/ionization (MALDI) time-of-flight (TOF) mass spectrometry (MS), and nanospray desorption electrospray ionization (nano-DESI) Orbitrap MS/MS (Supplementary Figs 4 and 5).

**Figure 3:**
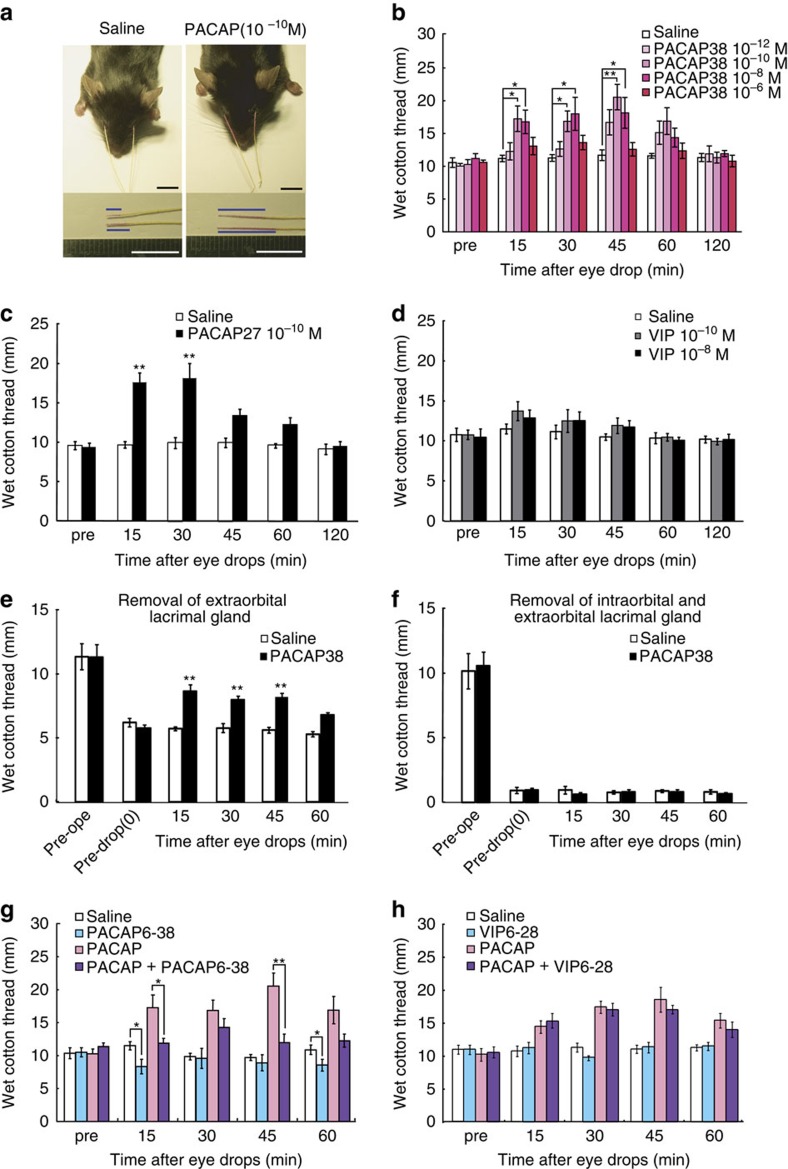
Effect of PACAP eye drops on tear secretion in male mice. (**a**) Representative images of the cotton thread measurements 30 min after administration of 10^−10^ M PACAP38 or saline eye drops with a 30 s insertion of the thread into both lower eyelids. The blue bar in the lower pictures shows the length of the tear-absorbed thread that changed colour from yellow to red. Scale bar, 10 mm. (**b**) Dose-response effect of PACAP38 eye drops on tear secretion in mice. Test solution eye drops were applied to both eyes. The sum of the wet length of cotton from the two eyes is shown on the *Y*-axis (*n*=10 per group). *P<0.05, **P<0.01. (**c**,**d**) Effect of PACAP27 (**c**) and VIP (**d**) eye drops on tear secretion (*n*=9–10 per group). **P<0.01 versus the saline-treated group. (**e**,**f**) Effect of 10^−10^ M PACAP38 eye drops on two lacrimal gland-deficient models: removal of the exorbital lacrimal gland (**e**) and removal of both the infraorbital and exorbital lacrimal glands (**f**) (*n*=10 per group). **P<0.01 versus the saline-treated group. (**g**,**h**) Effect of PAC1-R and VPAC2-R antagonist, PACAP6–38 (**g**) and VPAC1-R and VPAC2-R antagonist, VIP6–28 (**h**) treatments on PACAP-induced tear secretion (*n*=10 per group). *P<0.05, **P<0.01.

To investigate the function of PACAP in the lacrimal gland, PACAP38 was delivered in the form of eye drops to wild-type mice, and the level of tear secretion was measured using the cotton thread method (Fig. 3a). Eye drops containing 10^−10^ to 10^−8^ M PACAP38 significantly increased tear secretion from 15 to 45 min after treatment, with levels returning to baseline by 120 min (Fig. 3b). The basal tear secretion level and PACAP-induced tear secretion level did not differ significantly between males and females (Supplementary Fig. 6). PACAP27-containing eye drops also stimulated lacrimation, whereas the structurally related peptide VIP did not (Fig. 3c,d). Given that it has been reported that PACAP38, rather than PACAP27, is predominantly expressed in mammalian tissues^10^, PACAP38 was used in the following experiments. When PACAP was administered unilaterally, tear secretion was only induced on the PACAP-treated side (Supplementary Fig. 7). When corneas were pre-treated with the topical anaesthetic Benoxil to suppress the corneal reflex, the basal lacrimation level decreased, but PACAP still elicited a significant increase in tear secretion (Supplementary Fig. 8). PACAP can thus induce tear secretion under topical anaesthesia, showing that it has a direct effect on the infraorbital lacrimal gland in the absence of the corneal/conjunctival reflex. Moreover, when the acute to semi-acute toxicological effect of PACAP38 (10^−7^ M) eye drops was evaluated at a concentration 1,000 times higher than an effective dose of PACAP38 (10^−10^ M) 48 h after the eye drop treatment, no morphological changes were observed in the corneas or in the infraorbital lacrimal glands (Supplementary Fig. 9). In addition, we examined the effect of PACAP on angiogenesis *in vitro* using human endothelial cells and fibroblasts in co-culture systems. No changes were observed in either the PACAP38 or PACAP6–38 (a PAC1-R and VPAC2-R antagonist)-treated groups (Supplementary Fig. 10). These data suggest that PACAP eye drops act locally to stimulate lacrimation without causing acute to semi-acute toxicity or eliciting a corneal reflex. Mice have two lacrimal glands (the infraorbital and exorbital glands) (Supplementary Fig. 11a), both of which were found to express PAC1-R and VPAC1-R (Supplementary Fig. 11b). Although PACAP still significantly stimulated lacrimation in a mouse model in which the exorbital lacrimal gland had been removed, it could not stimulate tear secretion in a second model in which both lacrimal glands had been removed (Fig. 3e,f). These findings indicate that the target organ for PACAP administered in an eye drop formulation is the infraorbital lacrimal gland.

We next examined the signalling cascade associated with PACAP-induced lacrimation. Pre-treatment with PACAP6–38 significantly suppressed PACAP-induced tear secretion (Fig. 3g), whereas VIP6–28 (a VPAC1-R and VPAC2-R antagonist) did not suppress tear secretion (Fig. 3h). Moreover, a single drop of PACAP6–38 (10^−8^ M) to the ocular surface reduced the level of normal lacrimation at the 15 and 60 min time points post-administration (Fig. 3g). The intravenous infusion of PACAP also increased tear secretion in a manner that could be inhibited by co-treatment with PACAP6–38 (Supplementary Fig. 12). These results indicate that PACAP eye drops stimulate lacrimation via an action on PAC1-R.

### PACAP eye drops to *Adcyap1* ^*−/−*^ mice

**Figure 4:**
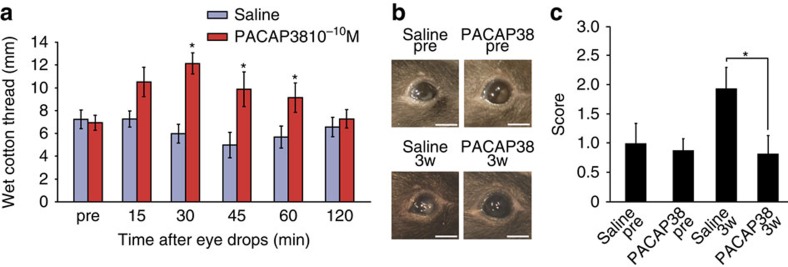
Effect of PACAP eye drops on female *Adcyap1*^*−/−*^ mice. (**a**) Tear secretion level in *Adcyap1*^*−/−*^ mice after application of saline or 10^−10^ M PACAP38 eye drops (*n*=9 per group). *P<0.05 versus saline-treated group. (**b**,**c**) Corneal scoring before and after the repeated application of PACAP eye drops to *Adcyap1*^*−/−*^ mice over a 3-week period. (**b**) Representative images of the corneal surface before and after eye drop application. Scale bar, 2 mm. (**c**) Eighteen female *Adcyap1*^*−/−*^ mice were given saline or PACAP eye drops unilaterally. The classification of each side of the cornea was evaluated separately before, and 3 weeks after eye drop treatment commenced. *P<0.05.

We also used PACAP-containing eye drops on *Adcyap1*^*−/−*^ mice. PACAP38 (10^−10^ M) drops increased tear secretion in these mice as well as in their wild-type counterparts, suggesting that PACAP transiently restores tear secretion in *Adcyap1*^*−/−*^ mice (Fig. 4a). We subsequently tested the effects of repeated administration of PACAP38 on the eyes of *Adcyap1*^*−/−*^ mice (one eye treated with PACAP38, the other with saline) with a view to preventing corneal keratinization. After 3 weeks of treatment, the injury score had increased in saline-treated eyes, but was still at the pre-treatment level in PACAP-treated eyes (Fig. 4b,c). Angiogenesis and ocular hyperaemia were not observed in PACAP-treated eyes (Fig. 4b).

### Signalling associated with PACAP-induced tear secretion

**Figure 5:**
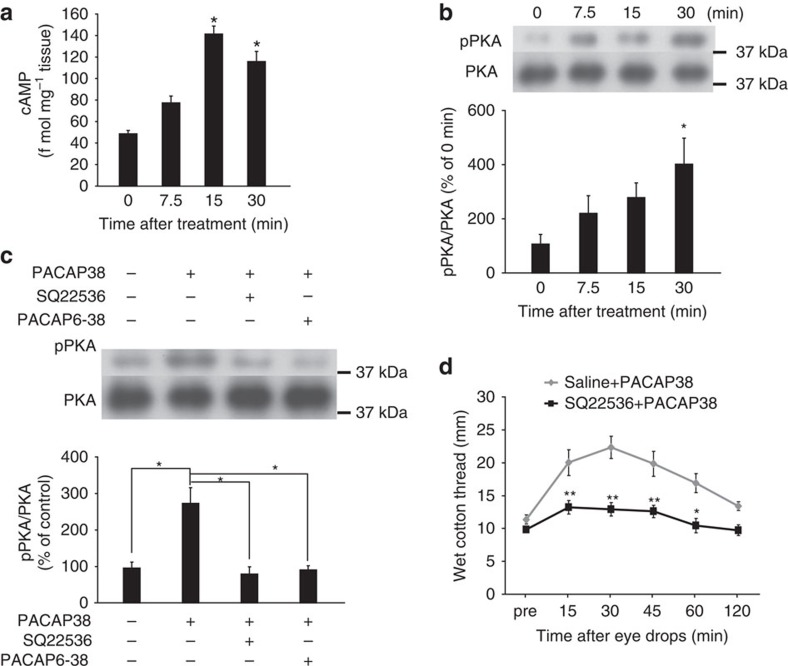
Contribution of the cAMP/PKA pathway to PACAP-induced tear secretion. (**a**) cAMP content in infraorbital lacrimal gland extracts after application of 10^−10^ M PACAP eye drops (*n*=8 per group). *P<0.05 versus 0 min. (**b**) Phosphorylated PKA (pPKA) levels in the mouse infraorbital lacrimal gland after PACAP eye drop application (*n*=6 per group). *P<0.05 versus 0 min. (**c**) Pre-treatment with the adenylate cyclase inhibitor SQ22536 or the PACAP receptor antagonist PACAP6–38 suppressed PKA phosphorylation 30 min after PACAP eye drop administration (*n*=6 per group). *P<0.05. (**d**) Effect of SQ22536 pre-treatment on PACAP-induced tear secretion in male mice (*n*=10 per group). A single drop of test solution was applied to each ocular surface. The sum of the wet lengths of thread from both eyes is shown. *P<0.05, **P<0.01 versus saline+PACAP group.

To determine the pathway related to PACAP-induced tear secretion, we next investigated the signalling pathways downstream of PAC1-R, focusing on the adenylate cyclase (AC)-cAMP-dependent pathway. As determined by ELISA, the cAMP level in mouse infraorbital lacrimal glands was increased at 15 min and peaked 30 min after the application of PACAP38-containing eye drops (Fig. 5a). The signal for phosphorylated (p) protein kinase A (PKA), a cAMP-dependent protein kinase, was significantly increased at 30 min (Fig. 5b), while pre-treatment with the AC inhibitor SQ22536 or with PACAP6–38 7.5 min before the administration of PACAP eye drops significantly suppressed the PACAP-induced phosphorylation of PKA (Fig. 5c). Pre-treatment with the AC inhibitor dramatically suppressed PACAP-induced tear secretion (Fig. 5d).

### Aquaporin expression in wild-type and *Adcyap1* ^*−/−*^ mice

**Figure 6:**
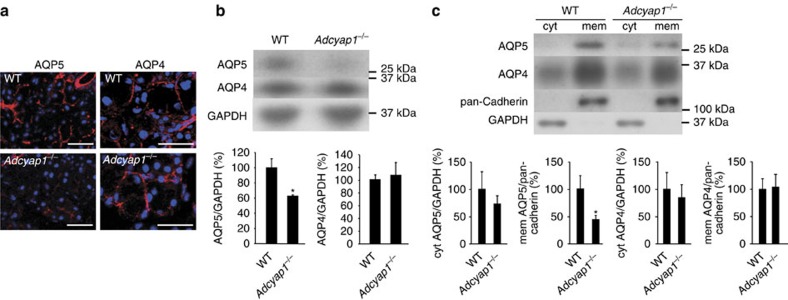
AQP4 and AQP5 levels in the infraorbital lacrimal glands of male wild-type and *Adcyap1*^*−/−*^ mice. (**a**) AQP4 and AQP5 immunoreactivities in wild-type and *Adcyap1*^*−/−*^ mouse infraorbital lacrimal glands. Scale bar, 50 μm. (**b**) AQP4 and AQP5 signals in infraorbital lacrimal gland total lysates from wild-type and *Adcyap1*^*−/−*^ mice detected by western blot analysis and expressed as semi-quantified results (*n*=6 per group). *P<0.05 versus wild-type mice. (**c**) AQP4 and AQP5 signals from fractionated infraorbital lacrimal glands and the semi-quantified results for wild-type and *Adcyap1*^*−/−*^ mice (*n*=6 per group). *P<0.05 versus wild type. GAPDH and pan-cadherin signals were used as internal controls for cytosolic and membrane protein, respectively.

The aquaporins (AQPs) are a family of water channel proteins that are expressed in numerous tissues and organs, with expression of the AQP4 and AQP5 subtypes being reported in the lacrimal gland^18^. To evaluate the relationship between PACAP-induced tear secretion and AQPs, we examined AQP4 and AQP5 immunoreactivities in the infraorbital lacrimal gland in wild-type and *Adcyap1*^*−/−*^ mice. AQP5 immunoreactivity was identified on the apical side of acinar cells in wild-type mice, but only weak immunoreactivity was observed in these cells in *Adcyap1*^*−/−*^ mice (Fig. 6a). AQP4 immunoreactivity was observed on the basal side of acinar cells in both wild-type and *Adcyap1*^*−/−*^ mice, without any obvious difference between the two (Fig. 6a). The specificity of the AQP4 and AQP5 immunoreactivities was confirmed using an antigen pre-absorption test for AQP5 antibody or comparison with a primary antibody-free (AQP4 antibody) negative control (Supplementary Fig. 3d,e). On immunoblotting, the AQP5 signal was found to be significantly lower in the infraorbital lacrimal glands of *Adcyap1*^*−/−*^ mice than in those of wild-type animals, but the AQP4 signal was almost the same in both cases (Fig. 6b).

The trafficking of AQP5 protein from the cytosol to the membrane contributes to increased water permeability^19^. After fractionation and immunoblotting, the AQP5 signal in the membrane fraction was significantly lower in *Adcyap1*^*−/−*^ mice than in wild-type mice, but was not significantly different in the cytosolic fraction (Fig. 6c). In contrast, the APQ4 signals in the cytosolic and membrane fractions were similar for the two groups (Fig. 6c).

### AQP5 expression and distribution after PACAP eye drops

**Figure 7 f7:**
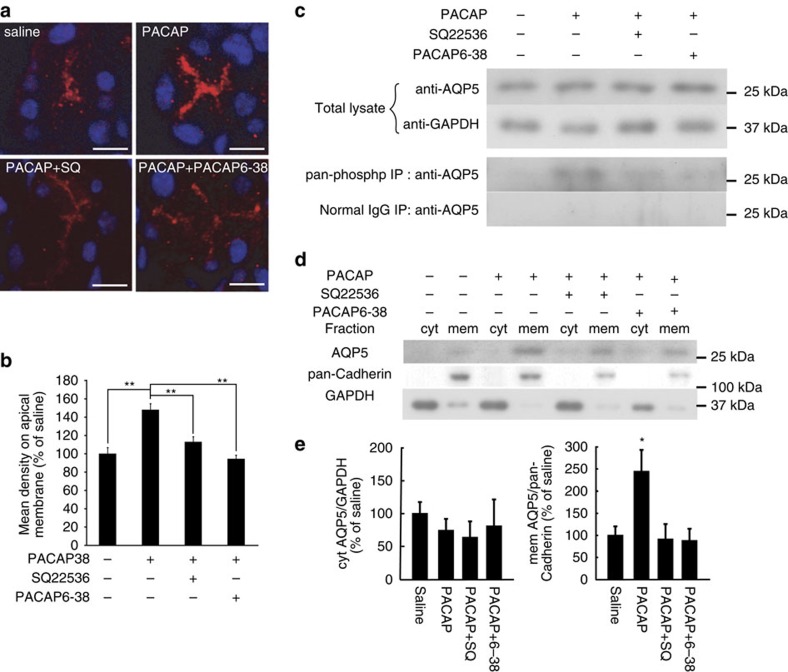
PACAP eye drop-induced phosphorylation and trafficking of AQP5. (**a**) Representative images of AQP5 immunoreactivities in infraorbital lacrimal gland acinar cells after the application of saline, PACAP38, PACAP38+SQ22536 (SQ) or PACAP38+PACAP6–38 eye drops. Scale bar, 10 μm. (**b**) AQP5 signals in fractionated infraorbital lacrimal glands and the semi-quantified results (*n*=11 per group). **P<0.01. (**c**) AQP5 signals in total lysate and immunoprecipitation with a pan-phospho antibody. Immunoprecipitation with a normal IgG antibody was used as a negative control. (**d**,**e**) AQP5 signals in fractionated infraorbital lacrimal glands (**d**) and semi-quantified results (**e**) (*n*=11 animals per group). *P<0.05

**Figure 8:**
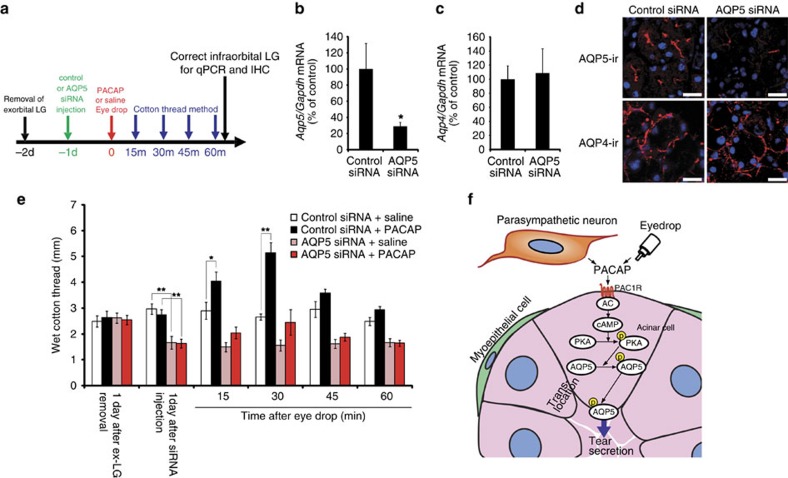
Contribution of AQP5 to PACAP-induced tear secretion in mice. (**a**) Experimental design of AQP5 siRNA study. (**b**–**d**) AQP4 and AQP5 levels in the infraorbital lacrimal glands of male wild-type mice 1 day after AQP5 siRNA treatment. *Aqp4* (**b**) and *Aqp5* (**c**) mRNA levels were measured by real-time PCR (*n*=12 per group). *P<0.05 (**d**) Representative pictures of AQP4 and AQP5 immunoreactivities in the infraorbital lacrimal gland after control and AQP5 siRNA injection. Scale bar, 20 μm. (**e**) Effect of AQP5 siRNA treatment of infraorbital lacrimal glands on PACAP-induced tear secretion (*n*=12). *P<0.05, **P<0.01. (**f**) Schema of proposed mechanisms of PACAP-induced tear secretion.

The phosphorylation of AQP5 has been postulated to initiate its trafficking to the membrane^20^,^21^. On this basis, the cellular localization and degree of phosphorylation of AQP5 was evaluated in PACAP-treated infraorbital lacrimal glands. Thirty minutes after treatment with 10^−10^ M PACAP, AQP5 immunoreactivity on the apical side of acinar cells was greater than that in the saline-treated, SQ22536-pre-treated or PACAP6–38-pre-treated groups (Fig. 7a,b). The AQP5 levels in the total lysates of the infraorbital lacrimal gland extracts showed no difference between the groups at 30 min (Fig. 7c). However, the AQP5 signal in infraorbital lacrimal gland extracts immunoprecipitated with a pan-phospho antibody was clearly detectable in the PACAP-treated group, but was less obvious in the other groups (Fig. 7c). An AQP4 signal was not detected in the sample immunoprecipitated with the pan-phospho antibody (Supplementary Fig. 13). The AQP5 signals in the membrane fractions were increased 30 min after treatment with PACAP compared with the other groups (Fig. 7d,e). To elucidate the contribution of AQP5 to PACAP-induced tear secretion, an AQP5 gene-silencing experiment was designed (Fig. 8a). When infraorbital lacrimal glands were treated with AQP5 siRNAs, the *Aqp5* mRNA level was significantly decreased by about 70%, whereas the *Aqp4* mRNA level remained almost the same 24 h after the siRNA treatment compared with the control group (Fig. 8b,c). AQP5 siRNA treatment reduced AQP5 immunoreactivity in the infraorbital lacrimal glands, but not AQP4 immunoreactivity (Fig. 8d). The AQP5 siRNA treatment significantly decreased the basal level of tear secretion, and PACAP-induced tear secretion was attenuated 24 h after the siRNA treatment (Fig. 8e).

Taken together, these findings suggest an underlying mechanism whereby PACAP and its receptor are expressed in mouse infraorbital lacrimal glands. PACAP stimulates tear secretion via an AC/cAMP/PKA cascade, which in turn stimulates AQP5 translocation from the cytosol to the membrane of lacrimal acinar cells to bring about an increase in water permeability (Fig. 8f).

## Discussion

Dry eye syndrome is more common in women than in men, particularly in older patients^2^. This study has made use of the discovery of a new *Adcyap1*^*−/−*^ mouse phenotype, namely the manifestation of corneal keratinization, which was particularly apparent in female mice as a function of age, the overall implication being that *Adcyap1*^*−/−*^ mice manifest dry eye-like signs. The tear-secreting response to PACAP eye drops and the basal level of tear secretion were, nevertheless, similar between male and female wild-type mice (Supplementary Fig. 6). It has been suggested that the sex difference in dry eye syndrome in humans is due to the influence of female sex hormone levels^22^. Given that female *Adcyap1*^*−/−*^ mice exhibit decreased serum progesterone levels^23^, dry eye-like symptoms in these animals may be due to an imbalance in their sex hormone levels.

Animal models that mimic dry eye disorder have been established by several groups^24^,^25^. One type of dry eye is the aqueous-deficient model, which mimics the dry eye symptoms caused by autoimmune diseases such as Sjögren's syndrome, removal or irradiation of the lacrimal gland, or neuronal pathway dysfunction. Another type is the evaporative dry eye model, as seen with meibomian gland dysfunction, environmental stress such as exposure to a dry environment, and pharmaceutically induced tear film instability caused by a decrease in oil or mucin secretion in the tears as evidenced by dry eye symptoms^1^. However, a dry eye model arising from a specific endocrine imbalance has not been reported to date. We have shown that *Adcyap1*^*−/−*^ mice exhibit (1) reduced lacrimation when left untreated, (2) increased lacrimation upon PACAP administration and (3) a morphologically normal lacrimal gland. These findings suggest that the impairment of lacrimal secretion in *Adcyap1*^*−/−*^ mice results from lacrimal gland dysfunction rather than developmental or structural abnormalities. Moreover, *Adcyap1*^*−/−*^ mice spontaneously develop corneal keratinization with aging, implying that this mouse phenotype could serve as a novel non-Sjögren's type aqueous-deficient dry eye model arising from lacrimal gland dysfunction.

AQP family genes and proteins are expressed in the eye and its accessory organs^26^,^27^. It has been reported that AQP5 immunoreactivity is markedly decreased in the lacrimal acinar cells of people with Sjögren's syndrome, a chronic autoimmune disorder with impairment of the moisture-producing glands^28^. The reduced expression of AQP5 suggests that this protein may be related to the decrease in tear secretion in this disease. On the other hand, Verkman's group reported that AQP5 KO mice do not exhibit an altered tear volume^29^. However, the same group recently published data showing that the Na^+^ content of tears from AQP5 KO mice is significantly higher than that of wild-type mice^30^, suggesting that the ion concentration in AQP5 KO mouse tears is elevated due to decreased water secretion into the tear fluid. In our study, AQP5 gene silencing-attenuated PACAP-induced tear secretion, as well as the basal level of tear secretion. Taken together, we propose that AQP5 is associated with water secretion into the tear.

It has been reported that the activation of cAMP/PKA can induce the translocation of AQP5 from the cytosol to the apical membrane^31^,^32^. Moreover, X-ray analysis of the structure of human AQP5 has revealed that phosphorylation at the C-terminal is required for the conformational change for trafficking^21^. Although the relationship between membrane trafficking and phosphorylation of AQP5 is not yet fully understood, in the case of AQP2, the closest paralog of AQP5, a key event for membrane trafficking is the phosphorylation of a C-terminal site by PKA (refs 33, 34). Here, we demonstrate that PACAP eye drops induce an elevation of cAMP, pPKA and pAQP5 levels and membrane trafficking of AQP5 in the mouse infraorbital lacrimal gland, suggesting that PACAP is a regulator of AQP5 trafficking in this gland.

Gilbard and collaborators reported that topical administration of a cAMP inducer such as VIP stimulates tear secretion in a rabbit model of keratoconjuctivitis sicca^35^ and in patients with dry eye disease^36^. However, they did not further study the nature of the target organ, or how reagents stimulate tear secretion. Here, we have demonstrated that PACAP stimulates tear secretion from the infraorbital lacrimal gland via a PAC1-R/cAMP/PKA/AQP5 cascade. Moreover, PACAP had a much greater effect on tear secretion than VIP. It should be noted that the Gilbard study used a high concentration of VIP (2 × 10^−6^ M) as eye drops in their rabbit model, whereas we used 10^−10^ M and 10^−8^ M, the same as our effective doses of PACAP in mice. We also found that PAC1-R is the main receptor for PACAP-induced tear secretion. The affinity of PACAP for PAC1-R is 1,000 times higher than that of VIP, implying that the higher concentration of VIP may be able to stimulate tear secretion via PAC1-R. Taken together, these results suggest that cAMP signalling is an important step for tear secretion, and that PACAP is an endogenous tear regulator in the lacrimal gland, with much greater potential than VIP to stimulate lacrimation.

Tear fluid includes several antibacterial proteins, growth factors and secretary mucin for corneal maintenance^37^,^38^. As it has been reported that systemic infusion of PACAP alters the composition of tears, especially the keratin family of proteins in rats^39^, PACAP may regulate tear protein secretion as well as tear fluid secretion. It is also well known that tear secretion is important for corneal healing^40^, and for this reason we postulated that a reduction in tear fluid would be an important factor underlying corneal keratinization in the *Adcyap1*^*−/−*^ mouse, and that PACAP could protect the corneal surface by stimulating tear secretion. Moreover, we used MALDI-TOF MS and nano-DESI MS/MS to identify the presence of PACAP in mouse tear fluid, a finding that may imply that PACAP is secreted from the lacrimal gland into the tear fluid, thereby directly affecting the cornea. However, it remains questionable from which tissues in tear fluid PACAP is derived. The PACAP could be coming from corneal and conjunctival nerve endings released by dromic (parasympathetic or sympathetic nerves) or anti-dromic (sensory nerves) stimulation, conjunctival goblet cells or stratified squamous cells, or the meibomian glands. If the corneal keratinization in *Adcyap1*^*−/−*^ mice is due to evaporative water loss caused by a reduced volume of tears, it could be postulated that lid closure (for example, reversible closure, using cyanoacrylate glue) in *Adcyap1*^*−/−*^ mice would inhibit the development of this phenotype. Future studies will be needed to clarify the source of PACAP in tear fluid and the corneal healing effects of PACAP.

Although our findings indicate the potential of PACAP as a stimulator of tear production in mice, there are still problems that would need to be resolved in relation to drug development. First, the human and mouse lacrimal apparatuses are structurally different. In mice, the exorbital lacrimal glands are located near the ear on the lateral side of the skull, and connect with the excretory ducts into the eyelid. The infraorbital lacrimal glands and the lipid-secreting Harderian glands are found in the orbit of the eye. In humans, one main lacrimal gland is located in the lacrimal fossa of each orbit next to the eye ball; this connects with excretory ducts in the upper fornix. However, over 50 accessory lacrimal glands are scattered over the inner surfaces of the lower and upper eyelids, and the lipid-secreting meibomian gland is located in the tarsal plate of the upper and lower eyelids^41^. We anticipate that PACAP eye drops will not reach the main lacrimal gland in humans because of its location, making the target of PACAP the accessory lacrimal glands. However, it is still an open question whether PACAP only affects the lacrimal gland, or whether the corneal and conjunctival epithelia, and the intraorbital lipid-secreting gland are also involved. In any future clinical trials of PACAP on dry eye patients, it will be important to clarify the expression of PACAP and PACAP receptors in tear-secreting tissue, including the lacrimal glands, as well as the effect and target of PACAP eye drops in humans. Moreover, we showed that PACAP eye drops did not cause any adverse reaction in acute to semi-acute phase at a concentration of 10^−7^ M (Supplementary Fig. 9), or in chronic eye drop treatment at a concentration of 10^−10^ M for 3 weeks in *Adcyap1*^*−/−*^ mice (Fig. 4). However, these toxicological evaluations may not be sufficient for safety trials of PACAP eye drops to proceed on healthy volunteers. Further suitable toxicological tests will be required before a clinical trial.

In this study, PACAP38 eye drops at the lower dose of 10^−10^ M stimulated mouse lacrimation for <1 h. Even if the effects of PACAP are similar in mice and humans, this short period of action would pose a problem in a clinical setting. We believe that the short-acting effect of PACAP in our study was due to the PACAP eye drops being washed from the ocular surface by lacrimation. One solution could be to formulate an ointment to provide sustained release. However, low doses of bioactive peptides have both advantages and disadvantages in terms of drug development. A low dose of peptide-based medicine offers good efficacy, safety and high selectivity and potency. In contrast, there are issues in relation to instability, short half-life and rapid elimination^42^. These problems would need to be overcome if PACAP were to be developed as an effective stimulator of lacrimation.

In conclusion, our results highlight a new function of PACAP as a stimulator of tear production initiated via the PAC1-R/AC/cAMP/PKA/AQP5 cascade. We found that PACAP eye drops induce tear secretion and suppress the progression of corneal keratinization in *Adcyap1*^*−/−*^ mice. Topical administration of cyclosporine has been developed for dry eye patients to provide an anti-inflammatory effect; however, eye drops focusing on tear-stimulating mechanisms are still in the developmental stage. The findings from our work are encouraging and should provide the impetus for further preclinical and clinical studies on the efficacy of PACAP eye drops to treat dry eye patients.

## Methods

### Animals

All experimental procedures involving animals were approved by the Institutional Animal Care and Use Committee of Showa University (08116, 09110, 50021, 51033, 510043, 52007, 52013, 53020, 53025). The *Adcyap1*^*−/−*^ mice on the C57BL/6 background were established by Dr Akemichi Baba^15^ and were bred and maintained under specific pathogen-free conditions in the animal facility of Showa University. Animals were housed in a facility with a 12-h/12-h light/dark cycle and were given free access to water and standard rodent chow. Eight to twelve weeks old mice were used, excepting Figs 1 and 4, and Supplementary Figs 1 and 2 with 8 to over 30 weeks old mice. In this study, both male and female mice were used as described in the figure legends.

### Evaluation of corneal scoring

Keratinization of the cornea was classified into four grades (Grades 0–3) by visual observation under a dissecting microscope as follows: Grade 0 (normal)=no observable abnormality; Grade 1=clouded cornea; Grade 2=angiogenesis; and Grade 3=hypertrophy of the corneal epithelium as shown in Supplementary Fig. 1a–d. The evaluation of corneal score was performed in a blinded fashion. The corneal keratinization of wild-type, *Adcyap1*^*+/−*^, and *Adcyap1*^*−/−*^ mice was evaluated on both sides, and the higher grade was used for the grading of the animals in Fig. 1e,f.

### Corneal fluorescein staining

Corneal injuries were visualized with fluorescein staining. *Adcyap1*^*−/−*^ and wild-type mice were anaesthetized with an intraperitoneal (i.p.) injection of pentobarbital, and 2 μl of fluorescein solution (1 μg μl^−1^) dissolved in saline were dropped on the cornea. After letting the animal blink five times, excess drops were wiped off with a cotton swab. Photographs were taken with a digital camera (CAMEDIA C5050, Olympus, Tokyo, Japan) connected to a stereoscopic microscope.

### PACAP-containing eye drops and cotton thread tear test

The rate of lacrimal secretion was determined with the cotton thread test using standardized phenol red-impregnated cotton threads (Zone-Quick; Menicon Co. Ltd, Nagoya, Japan). Mice were anaesthetized with an i.p. injection of pentobarbital to suppress autonomic nervous system reflexes that could have affected tear secretion. The basal level of tear secretion was evaluated by insertion of the thread under the lower eyelid for 30 s, after which the bilateral length of the colour-changed thread that had absorbed the tear fluid was measured in millimeters. The test solution, PACAP38 (10^−6^–10^−12^ M (Peptide Institute, Osaka, Japan), PACAP27 (10^−10^ M, Peptide Institute), VIP (10^−8^ M or 10^−10^ M, Peptide Institute) or saline was then applied to both ocular surfaces. The mice were forced to blink their eyes five times, and their eyes were then kept closed during all subsequent steps to prevent drying of the surface of the cornea.

Excess drops were wiped off with a swab 7.5 min after application of the eye drops, and tear secretion was checked at 15-min intervals from 15 to 120 min after the eye drops had been administered. At each measurement time, the thread was kept on the lower conjunctival sac of each eye for 30 s. The tear volume in the no eye drop study (Fig. 1; Supplementary Fig. 12) was performed without anaesthesia. The total length of wet cotton thread was measured for both sides (Figs 1d,e, 3, 4 and 5;; Supplementary Figs 8 and 12), and in other specific experiments (Figs 1f,g and 8; Supplementary Fig. 7), the length was evaluated on each side. The PACAP receptor antagonist PACAP6–38 (Peptide Institute), VIP receptor antagonist VIP6–28 (Sigma, St Louis, MO, USA), adenylate cyclase inhibitor SQ22536 (Sigma-Aldrich) or topical anaesthetic 0.4% oxybuprocaine hydrochloride (Benoxil; Santen Pharmaceutical Co., Ltd., Osaka, Japan) was applied 7.5 min before the administration of eye drops containing 10^−10^ M PACAP38 or saline. When Benoxil was pre-administered, the loss of the corneal reflex was confirmed by stimulating the corneal surface with a blunt plastic tip before PACAP or saline treatment.

### Histology

Adult C57BL/6 mice (Charles River Japan, Kanagawa, Japan) were anaesthetized with sodium pentobarbital (50 mg kg^−1^, i.p.) and perfused with phosphate-buffered saline (PBS) followed by 2% paraformaldehyde in PBS. Immediately afterwards, the infraorbital lacrimal glands were removed and fixed in the same fixative for 24 h at 4 °C. Fixed tissues were embedded in paraffin for histopathological studies with hematoxylin and eosin staining, or frozen sections were used for double-labelling immunofluorescence staining.

### Toxicology of the PACAP eye drops

In acute to semi-acute toxicology experiments, 2 μl of 10^−7^ M PACAP or saline were applied as eye drops, and the eye ball and accessory organs of treated animals were fixed with formaldehyde 48 h later. The acute to semi-acute toxicological effects of PACAP on the cornea and infraorbital lacrimal gland were evaluated based on hematoxylin and eosin staining.

### Double-labelling immunofluorescent staining

The following primary antibodies were used according to standard protocols^43^. Eight-μm-thick frozen sections were blocked in 5% normal horse serum for 1 h and incubated overnight at 4 °C in mixtures of the following primary antibodies: rabbit anti-PACAP antibody (1:2000; Peninsula Laboratories, Inc., Belmont, CA, USA), rabbit anti- PAC1-R antibody (1:200; raised by using the N-terminal residue as an antigen^44^), mouse anti-smooth muscle actin antibody (1:200; R&D Systems, Inc. Minneapolis, MN, USA), mouse anti-NeuN antibody (1:1000; Millipore, Billerica, MA, USA), goat anti-ChAT antibody (1:100; Millipore), rabbit anti-AQP4 antibody (1:250; Millipore) and rabbit anti-AQP5 antibody (1:100; Millipore). Immunoreactivity was detected using an Alexa 488- or 546-labelled goat anti-mouse IgG, goat anti-rabbit IgG or donkey anti-goat IgG antibodies (1:800; Invitrogen) following a 90-min incubation at room temperature. After washing with PBS, the sections were incubated for 5 min with 4',6-diamidine-2-phenylindole dihydrochloride (DAPI, 1:10,000; Roche Diagnostics, Indianapolis, IN, USA) to stain cell nuclei. Labelling was imaged with a fluorescence microscope (Axio Imager Z1 with Apotome, Carl Zeiss, Oberkochen, Germany). In the antigen absorption test, PACAP, PAC1-R or AQP5 antibody solution was incubated with a final concentration of 1 μM of antigens in 5% normal horse serum in PBS overnight at 4 °C. After centrifugation at 10,000*g* for 20 min, the supernatant was used instead of the primary antibody.

### Reverse transcriptase PCR

Total RNA was isolated from mouse infraorbital lacrimal glands with an RNAeasy kit (Qiagen, Hilden, Germany). Reverse transcription was performed with random primers. Mouse *Adcyap1* primers, forward; 5′-ATG ACC ATG TGT AGC GGA GCA AGG CTG G-3′, reverse; 5′-CTA CAA GTA TGC TAT TCG GCG TCC-3′ (product size 525 bp), and mouse *Adcyap1r1* primers, forward; 5′-CCT GTC GGT GAA GGC CCT CTA CAC A-3′, reverse; 5′- CCC AGC CCA AGC TCA AAC ACA AGT C-3′ (798–801 bp product for the hip or hop form, and 717 bp product for the short form), mouse *Vpacr1* primers, forward; 5′- GTA TGG ATG AGC AGC AAC AGA CTG-3′, reverse; 5′- TTG ATG ATG GTG TCC CAG CAC-3′ (product size 487 bp), mouse *Vpacr2* primers, forward; 5′-CAT CTC TGT GCT GGT CAA GGA C-3′, reverse; 5′- CGC CAT CTT CTT TTC AGT TCA C-3′ (product size 654 bp), mouse *Gapdh* primers, forward; 5′- GCC AAG GTC ATC CAT GAC AAC-3′, reverse; 5′-GTC CAC CAC CCT GTT GCT GTA-3′ (product size 498 bp), and mouse *Actb* primers, forward; 5′-GTG GGC CGC TCT AGG CAC CAA-3′, reverse; 5′-CTC TTT GAT GTC ACG CAC GAT TTC-3′ (product size 540 bp) were mixed with the EX taq kit (TAKARA Bio, Otsu, Japan) with the PCR conditions of penetration for 15 s at 95 °C, annealing at 50 °C for 30 s, elongation at 72 °C for 1 min with a final extension at 72 °C for 10 min. The PCR products were separated by gel electrophoresis on an agarose gel and visualized with an illuminometer. Image of the gel have been cropped for presentation. The uncropped images in Fig. 2a are shown in Supplementary Fig. 14.

### Real-time PCR

RNA isolation was followed by the reverse transcriptase PCR method. Reverse transcription into complementary DNA was achieved using the reagents and protocol of the PrimeScript RT reagent kit (TaKaRa BIO, Kyoto, Japan). The PCR primer set was as follows: mouse *Aqp4* primers, forward; 5′-CTT TCT GGA AGG CAG TCT CAG-3′, reverse; 5′-CCA CAC CGA GCA AAA CAA AGA T-3′, mouse *Aqp5* primers, forward; 5′-GGG TAA CCT GGC CGT CAA TG-3′, reverse; 5′-TGA CCG ACA AGC CAA TGG ATA A-3′, and mouse *Gapdh* primers, forward; 5′-TGT GTC CGT CGT GGA TCT GA-3′, reverse; 5′-TTG CTG TTG AAG TCG CAG GAG-3′. Real-time PCR was performed using SYBR Premix Ex Taq II reagent (TaKaRa BIO Inc.) and an ABI PRISM 7900 instrument (Applied Biosystems, Lincoln, CA, USA). Relative gene expression was calculated using the comparative delta Ct method with the Ct values of the housekeeping gene, *Gapdh*. mRNA levels were normalized, with the percentage for control groups taken as 100%.

### Repeated eye drop study

*Adcyap1*^*−/−*^ female mice were given eye drops containing 10^−10^ M PACAP or saline, 2 μl per eye (unilaterally), 2 times per day, 6 days per week for 3 weeks. We selected 18 female *Adcyap1*^*−/−*^ mice based on similar average pre-treatment corneal keratinization scores on both sides. The scoring method was the same as the previous evaluation for each side separately.

### cAMP enzyme immunoassay

Enzyme-linked immunoassays for cAMP were performed using a cAMP enzyme immunoassay (EIA) kit (Cayman Chemicals, Grand Rapids, MI, USA) following the manufacturer's instructions. In brief, C57/BL6 mice were anaesthetized, and 2 μl of 10^−10^ M PACAP was applied to both ocular surfaces. Both infraorbital lacrimal glands were removed at 0, 7.5, 15 or 30 min after the application of the eye drops (*n*=8). They were then homogenized in 200 μl of 5% trifluoroacetic acid on ice. After centrifugation at 1,500*g* for 10 min, the lysate was mixed with 1 ml of ether for 10 s. After removal of the ether, the aqueous layer was acetylated according to the EIA kit manufacturer's instructions and used for the EIA assay. The absorption in each well was measured with a plate reader (POLARstar Omega; BMG LABTECH GmbH, Offenburg, Germany).

### Immunoblotting

For western blot analysis, mice were euthanized by decapitation and their infraorbital lacrimal glands were immediately removed. The infraorbital lacrimal glands were then homogenized in cold lysis buffer (10 mM Tris-HCl, 0.15 M NaCl and 1% Triton X-100) with a protease inhibitor cocktail (Sigma). Homogenates were centrifuged at 12,000*g* for 30 min at 4 °C and the resultant supernatant was collected as the total cell lysate, which was subsequently diluted with SDS sample buffer (250 mM Tris-HCl (pH 6.8), 4% SDS, 40% glycerol, 4% 2-mercaptoethanol and 0.002% bromophenol blue) and incubated for 12 h at 4 °C. This lysate sample (30 μg) was electrophoresed on a 7.5% polyacrylamide gel containing 0.1% SDS at 100 V. The protein bands were then transferred from the gel to polyvinylidinene fluoride membranes (Bio-Rad Laboratories, Inc. Hercules, CA, USA) for 3 h at 100 mA. The membrane was initially blocked with 2% Blockace (Dainihon Pharmaceutics, Osaka, Japan) in Tris- buffered saline with Tween 20 (TBS-T) for 1 h at room temperature and probed overnight with a mouse monoclonal antibody for PKA (1:1,000, Cell Signaling Technology, Danvers, MA), pPKA (1:1,000, Cell Signaling Technology), AQP4 (1:2,000, Millipore), AQP5 (1:4,000, Millipore), GAPDH (1:2,000, Millipore), or pan-cadherin (1:4,000, Abcam, Cambridge, UK) at 4 °C. After incubation with a sheep anti-mouse IgG HRP-conjugated antibody (1:2,000; GE Healthcare Bioscience, Little Chalfont, UK) for 1 h at room temperature, protein bands were revealed using a SuperSignal West Dura Extended Duration Substrate (Thermo Fisher Scientific Pierce Biotechnology, Rockford, IL, USA) and exposed onto X-ray Film. Subcellular protein fractionation (cytosolic and membrane fractions) was performed using the ProteoExtract Subcellular Proteome Extraction Kit according to the manufacturer's instructions (Calbiochem, Hessen, Darmstadt, Germany). The fractions were precipitated using a ProteoExtract Protein Precipitation Kit (Calbiochem). Precipitated protein was then dissolved in lysis buffer and the protein content was measured. Equivalent amounts of proteins (5 μg) for each fraction of subcellular fractionation were used for immunoblotting. GAPDH and pan-cadherin signals were used as the internal controls for cytosolic and membrane proteins, respectively. Image of the membrane have been cropped for presentation. The uncropped images in Figs 5, 6, 7 are shown in Supplementary Fig. 14.

### Immunoprecipitation

All immunoprecipitations were carried out using the immunoprecipitation kit Catch and Release v2.0 (Millipore) following the manufacturer's instructions. In brief, mouse infraorbital lacrimal gland lysate was obtained 30 min after the application of eye drops. Four-hundred microgram protein from the cell lysate, 1 μg of a mouse anti-pThy, -pSer, or -pThr (pan-phospho) IgG antibody (AnaSpec Inc, San Jose, CA, USA) for the capture antibody or a normal mouse IgG (Millipore) as the control antibody, and 10 μl of the antibody capture affinity ligand (total 500 μl) were mixed and placed in a Catch and Release v2.0 spin column containing prepacked immunprecipitation capture resin. After end-over-end shaking for 16 h at 4 °C, the column was centrifuged, washed 3 times and then eluted with 70 μl of the elution buffer. The eluent was analysed by immunoblotting.

### Removal of lacrimal glands

Under inhalation anaesthesia with sevoflurane, the hair from under the ear to the outer canthus of the eyes was shaved, and a 15 mm incision was made. In the exorbital lacrimal gland removal model, the exorbital lacrimal gland, which is located under the ear, was exposed and removed. In the infraorbital and exorbital lacrimal gland removal model, the exorbital lacrimal gland and the lacrimal duct were first isolated. The infraorbital lacrimal gland was exposed by pulling the lacrimal duct, after which both lacrimal glands were removed. The skin was then sutured and the animals were kept warm during their recovery from anaesthesia. The tear secretion level was checked before and after surgery.

### Semi-quantification of AQP5 immunoreactivity in lacrimal acini

The densities of AQP5 immunoreactivity in the mouse infraorbital lacrimal gland 30 min after administration of eye drops containing saline, PACAP38, PACAP38+SQ22536, or PACAP38+PACAP6–38 was evaluated. AQP5 immunostaining was performed following the above method. One hundred pictures of acini from 10 infraorbital lacrimal glands (10 acinus pictures/lacrimal gland) from five wild-type mice in each group were cut out with grey scale. Using Image J software (ver. 1.44p), the average density in the apical membrane, and in four spots of 1 μm^2^ in the cytosolic area were measured. The value of the apical membrane density was determined using the cytosolic density value as background. The quantification was performed with a blinded test, masking sample data for another person who used image J.

### AQP5 siRNA treatment *in vivo*

An HPLC grade of non-target negative control and three types of mouse AQP5 siRNAs (sequence was shown in Supplementary Table 1) were designed and purchased from BONAC Corporation (Fukuoka, Japan). Mouse AQP5 siRNAs (10 μM × 3 siRNAs) or negative control siRNA (30 μM) were mixed with an equal volume of atelogene local use (KOKEN, Tokyo, Japan) and gently incubated for 1 h at 4 °C. One day after the removal of the exorbital lacrimal gland, the siRNA was applied to surround the infraorbital lacrimal gland. AQP5 siRNA and control siRNA were used on opposite sides. The next day, mice were anaesthetized with pentobarbital, after which PACAP38 (10^−10^ M) or saline eye drops were administered. At the 15, 30, 45 and 60 min time points, the tear secretion level was measured using the cotton thread method. The infraorbital lacrimal gland was then removed and the AQP4 and AQP5 levels were checked by real-time PCR and immunostaining.

### Whole-cornea immunostaining

After euthanasia, the epithelial layer of the cornea was scraped off under a stereoscopic microscope. The eye ball was excised, then fixed in 4% paraformaldehyde for 2 h at room temperature. The cornea was dissected and washed 3 times with phosphate-buffered saline (PBS), after which it was incubated with blocking buffer (1% Triton X-100 and 10% normal horse serum in PBS) for 1 h. The cornea was then incubated with primary antibody (Neurofilament 200, 1:500, Sigma, St Louis, MO) diluted in blocking buffer for 3 days at 4 °C, washed four times in PBS, and incubated with a secondary antibody (alexa546-labelled anti-rabbit IgG, 1:500) diluted in the blocking buffer for 4 h at room temperature. After final washing in PBS, the cornea was mounted surface side up on a glass slide with aqueous mounting medium. Labelling was imaged with a fluorescence microscope (Axio Imager Z1 with Apotome, Carl Zeiss, Oberkochen, Germany).

### Dot blotting

PACAP38 or VIP (1 μl of 0.2 to 25 pmol μl^−1^) was dropped on a nitrocellulose membrane. After drying, the membrane was washed in Tris buffered saline with Tween 20 and blocking buffer, followed by immunoblotting as described above. A primary anti-PACAP antibody (1:4,000; Peninsula Laboratories, Belmont, CA, USA) was used in the dot blotting study.

### MALDI-TOF mass spectrometry of intact PACAP38 in tear samples

Tear samples were collected from mice by application of sterile filter paper strips (*n*=5, Schirmer paper), and PACAP38 was measured using MALDI-TOF mass spectrometry. Aqueous solutions of the standard and tear samples were loaded onto the target plate (MTP 384 massive target T, Bruker Daltonics, Bremen, Germany) by mixing 1.0 μl of each solution with the same volume of a saturated matrix solution, prepared fresh every day by dissolving α-cyano-4-hydroxycinnamic acid in acetonitrile/0.1% trifluoroacetic acid (1/2, v/v). The mass spectrometer used in this work was an Autoflex II TOF/TOF (Bruker Daltonics) operated in the linear mode. Ions were accelerated under delayed extraction conditions (140 ns) in the positive ion mode with an acceleration voltage of 20.00 kV. The instrument uses a 337 nm pulsed nitrogen laser, model MNL-205MC (LTB Lasertechnik Berlin GmbH, Berlin, Germany). External calibration was performed in each case using the average masses of the Bruker Peptide Calibration Standard (#206195, Bruker Daltonics). Protein masses were acquired within a range of 1,000–8,000 *m*/*z*. Each spectrum was produced by accumulating data from 800 consecutive laser shots. Bruker FlexControl 2.4 software was used for control of the instrument and Bruker FlexAnalysis 2.4 software for spectrum evaluation.

### Nanospray desorption electrospray ionization Orbitrap MS/MS analyses of PACAP38 in tear samples

Nanospray desorption electrospray ionization (nano-DESI) was used to acquire MS/MS spectra directly from Schirmer paper containing tears (*n*=6). The nano-DESI probe consisted of two fused silica capillaries (ID 50 μm, OD 150 μm, Polymicro Technologies, Molex, Lisle, IL) positioned at an angle to each other^45^. A solvent, consisting of methanol/water (9/1, v/v) with 2% formic acid, was propelled, at 0.5 μl min^−1^, through the primary capillary, forming a liquid bridge to the secondary capillary. The secondary capillary transported the solvent to the mass spectrometer inlet for nanospray ionization. The filter paper was soaked with 10 μl 0.1% trifluoroacetic acid (99%, Sigma-Aldrich) on a regular glass slide. The glass slide was placed on a motorized *x*,*y*,*z*-stage (Newport Corporation, Irvine, CA, USA) to position the sample under the nano-DESI probe^46^,^47^. Material was extracted from the wet surface of the Schirmer paper by the nano-DESI probe and analysed using a QExactive Plus Orbitrap mass spectrometer (Thermo Fisher Scientific, Bremen, Germany). The instrument mass resolving power was set to 70,000 (*m*/Δ*m*) and a high voltage of 3 kV was applied to the primary capillary. Selective ion monitoring was set to *m/z* 648.5±2, corresponding to PACAP38 (*z*=7) and tandem mass spectrometry was performed at *m/z* 648.5±1 Da, using higher energy collision-induced dissociation with a normalized collision energy of 20 applied. The same settings were used for the PACAP38 standard, wild-type mouse samples and PACAP^−/−^ mouse samples.

### Effect of PACAP on angiogenesis

The experiments on endothelial cell tube formation were conducted in 24-well dishes using an angiogenesis kit (Kurabo, Okayama, Japan), according to the manufacturer's instructions. Human umbilical vein endothelial cells and fibroblasts were co-cultured in medium containing vascular endothelial growth factor (final 10 μg l^−1^) with various concentrations of PACAP38 (10^−9^, 10^−6^ M) and PACAP6–38 (10^−8^, 10^−6^ M), with the medium exchanged on days 4, 7 and 9. On day 11, the cells were washed and directly fixed in the wells with 70% ice-cold ethanol for 30 min. The fixed cells were serially incubated with 1% bovine serum albumin (BSA) in the buffer, a mouse monoclonal antibody against human CD31, an alkaline phosphatase-conjugated goat anti-mouse IgG, and a nitro-blue tetrazolium chloride (NBT)/5-bromo-4-chloro-3′-indolylphosphatase p-toluidine salt from the kit, and then washed and photographed. The images were analysed using Angiogenesis Image Analyzer software (Kurabo) to measure the gross area of the CD31-positive tubes (the area of endothelial tubes) and the length of CD31-positive tubes in culture. Data are shown as a percentage of the area of the endothelial cell tubes in the untreated cultures.

### Systemic infusion of PACAP38

Systemic infusion of PACAP was done as in our previous study^48^. Briefly, PACAP38 (5 nmol kg^−1^) or PACAP38 plus PACAP6–38 (50 nmol kg^−1^) was injected into the jugular vein with vehicle (0.1% BSA in saline) under inhalation anaesthesia with sevoflurane. A PE10 polyethylene tube connected to an Alzet osmotic pump (0.5 μl h^−1^; DURECT Corporation, Cupertino, CA, USA) that was filled with the vehicle, PACAP38 (32 pmol μl^−1^), or PACAP plus PACAP6–38 (320 pmol μl^−1^) was then inserted for continuous administration. Tear volume was measured with the cotton thread method four days after the infusion commenced.

### Statistical analyses

Data are presented as the mean±s.e. (*n*=sample size). The effects of treatments were analysed with the Mann–Whitney *U*-test (Figs 1i,j and 4c), the two-tailed Student's *t*-test (Figs 6b,c and 8b,c), one-way ANOVA followed by the Dunnett test (Figs 3b, 5a–c and Fig. 7e) or a one-way ANOVA followed by the Tukey test (Figs 1g,h, 3c–h, Figs 5d and7b, [Fig f7] and Fig. 8e and Supplementary Figs 6–8, 10 and 12). The two-tailed Spearman's correlation test was used to identify correlations between corneal grade and tear volume in *Adcyap1*^*−/−*^ mice. Differences due to the treatments were considered as significant for values of *P*<0.05.

### Data availability

The authors declare that the data supporting the findings of this study are available within the article and its Supplementary Information Files.

## Additional information

**How to cite this article:** Nakamachi, T. *et al.* PACAP suppresses dry eye signs by stimulating tear secretion. *Nat. Commun.* 7:12034 doi: 10.1038/ncomms12034 (2016).

## Supplementary Material

Supplementary Information Supplementary Figures 1-14 and Supplementary Table 1

## References

[b1] The definition and classification of dry eye disease: report of the Definition and Classification Subcommittee of the International Dry Eye WorkShop (2007). Ocul. Surf. 5, 75–92 (2007). 17508116 10.1016/s1542-0124(12)70081-2

[b2] Kastelan S., Tomic M., Salopek-Rabatic J. & Novak B. Diagnostic procedures and management of dry eye. Biomed. Res. Int. 2013, 309723 (2013). 24024186 10.1155/2013/309723 PMC3760183

[b3] Blehm C., Vishnu S., Khattak A., Mitra S. & Yee R. W. Computer vision syndrome: a review. Surv. Ophthalmol. 50, 253–262 (2005). 15850814 10.1016/j.survophthal.2005.02.008

[b4] Chen Q. *et al.* Lower volumes of tear menisci in contact lens wearers with dry eye symptoms. Invest Ophthalmol. Vis. Sci. 50, 3159–3163 (2009). 19279314 10.1167/iovs.08-2794

[b5] O'Brien P. D. & Collum L. M. Dry eye: diagnosis and current treatment strategies. Curr. Allergy Asthma Rep. 4, 314–319 (2004). 15175147 10.1007/s11882-004-0077-2

[b6] Miyata A. *et al.* Isolation of a neuropeptide corresponding to the N-terminal 27 residues of the pituitary adenylate cyclase activating polypeptide with 38 residues (PACAP38). Biochem. Biophys. Res. Commun. 170, 643–648 (1990). 2383262 10.1016/0006-291x(90)92140-u

[b7] Miyata A. *et al.* Isolation of a novel 38 residue-hypothalamic polypeptide which stimulates adenylate cyclase in pituitary cells. Biochem. Biophys. Res. Commun. 164, 567–574 (1989). 2803320 10.1016/0006-291x(89)91757-9

[b8] Harmar A. J. *et al.* Pharmacology and functions of receptors for vasoactive intestinal peptide and pituitary adenylate cyclase-activating polypeptide: IUPHAR review 1. Br. J. Pharmacol. 166, 4–17 (2012). 22289055 10.1111/j.1476-5381.2012.01871.x PMC3415633

[b9] Spengler D. *et al.* Differential signal transduction by five splice variants of the PACAP receptor. Nature 365, 170–175 (1993). 8396727 10.1038/365170a0

[b10] Vaudry D. *et al.* Pituitary adenylate cyclase-activating polypeptide and its receptors: 20 years after the discovery. Pharmacol. Rev. 61, 283–357 (2009). 19805477 10.1124/pr.109.001370

[b11] Nakamachi T. *et al.* Role of PACAP in neural stem/progenitor cell and astrocyte—from neural development to neural repair. Curr. Pharm. Des. 17, 973–984 (2011). 21524256 10.2174/138161211795589346

[b12] Shioda S. & Nakamachi T. PACAP as a neuroprotective factor in ischemic neuronal injuries. Peptides 72, 202–207 (2015). 26275482 10.1016/j.peptides.2015.08.006

[b13] Gonzalez-Rey E., Varela N., Chorny A. & Delgado M. Therapeutical approaches of vasoactive intestinal peptide as a pleiotropic immunomodulator. Curr. Pharm. Des. 13, 1113–1139 (2007). 17430175 10.2174/138161207780618966

[b14] Sherwood N. M., Adams B. A., Isaac E. R., Wu S. & Fradinger E. A. Knocked down and out: PACAP in development, reproduction and feeding. Peptides 28, 1680–1687 (2007). 17467121 10.1016/j.peptides.2007.03.008

[b15] Hashimoto H. *et al.* Altered psychomotor behaviors in mice lacking pituitary adenylate cyclase-activating polypeptide (PACAP). Proc. Natl Acad. Sci. USA 98, 13355–13360 (2001). 11687615 10.1073/pnas.231094498 PMC60875

[b16] Reglodi D. *et al.* PACAP is an endogenous protective factor-insights from PACAP-deficient mice. J. Mol. Neurosci. 48, 482–492 (2012). 22528455 10.1007/s12031-012-9762-0

[b17] Elsas T., Uddman R. & Sundler F. Pituitary adenylate cyclase-activating peptide-immunoreactive nerve fibers in the cat eye. Graefes Arch. Clin. Exp. Ophthalmol. 234, 573–580 (1996). 8880156 10.1007/BF00448802

[b18] Ishida N., Hirai S. I. & Mita S. Immunolocalization of aquaporin homologs in mouse lacrimal glands. Biochem. Biophys. Res. Commun. 238, 891–895 (1997). 9325187 10.1006/bbrc.1997.7396

[b19] Ishikawa Y. *et al.* Identification of AQP5 in lipid rafts and its translocation to apical membranes by activation of M3 mAChRs in interlobular ducts of rat parotid gland. Am. J. Physiol. Cell Physiol. 289, C1303–C1311 (2005). 16107506 10.1152/ajpcell.00211.2005

[b20] Woo J. *et al.* Membrane trafficking of AQP5 and cAMP dependent phosphorylation in bronchial epithelium. Biochem. Biophys. Res. Commun. 366, 321–327 (2008). 18042467 10.1016/j.bbrc.2007.11.078

[b21] Horsefield R. *et al.* High-resolution x-ray structure of human aquaporin 5. Proc. Natl Acad. Sci. USA 105, 13327–13332 (2008). 18768791 10.1073/pnas.0801466105 PMC2533189

[b22] Truong S., Cole N., Stapleton F. & Golebiowski B. Sex hormones and the dry eye. Clin. Exp. Optom. 97, 324–336 (2014). 24689906 10.1111/cxo.12147

[b23] Isaac E. R. & Sherwood N. M. Pituitary adenylate cyclase-activating polypeptide (PACAP) is important for embryo implantation in mice. Mol. Cell Endocrinol. 280, 13–19 (2008). 17945412 10.1016/j.mce.2007.09.003

[b24] Barabino S. & Dana M. R. Animal models of dry eye: a critical assessment of opportunities and limitations. Invest. Ophthalmol. Vis. Sci. 45, 1641–1646 (2004). 15161821 10.1167/iovs.03-1055

[b25] Schrader S., Mircheff A. K. & Geerling G. Animal models of dry eye. Dev. Ophthalmol. 41, 298–312 (2008). 18453777 10.1159/000131097

[b26] Castle N. A. Aquaporins as targets for drug discovery. Drug Discov. Today 10, 485–493 (2005). 15809194 10.1016/S1359-6446(05)03390-8

[b27] Verkman A. S. Role of aquaporin water channels in eye function. Exp. Eye Res. 76, 137–143 (2003). 12565800 10.1016/s0014-4835(02)00303-2

[b28] Tsubota K., Hirai S., King L. S., Agre P. & Ishida N. Defective cellular trafficking of lacrimal gland aquaporin-5 in Sjogren's syndrome. Lancet 357, 688–689 (2001). 11247557 10.1016/S0140-6736(00)04140-4

[b29] Moore M., Ma T., Yang B. & Verkman A. S. Tear secretion by lacrimal glands in transgenic mice lacking water channels AQP1, AQP3, AQP4 and AQP5. Exp. Eye Res. 70, 557–562 (2000). 10870513 10.1006/exer.1999.0814

[b30] Ruiz-Ederra J., Levin M. H. & Verkman A. S. In situ fluorescence measurement of tear film [Na+], [K+], [Cl−], and pH in mice shows marked hypertonicity in aquaporin-5 deficiency. Invest. Ophthalmol. Vis. Sci. 50, 2132–2138 (2009). 19136711 10.1167/iovs.08-3033 PMC2904304

[b31] Kosugi-Tanaka C. *et al.* Protein kinase A-regulated membrane trafficking of a green fluorescent protein-aquaporin 5 chimera in MDCK cells. Biochim. Biophys. Acta 1763, 337–344 (2006). 16603260 10.1016/j.bbamcr.2006.02.005

[b32] Yang F., Kawedia J. D. & Menon A. G. Cyclic AMP regulates aquaporin 5 expression at both transcriptional and post-transcriptional levels through a protein kinase A pathway. J. Biol. Chem. 278, 32173–32180 (2003). 12783871 10.1074/jbc.M305149200

[b33] Fushimi K., Sasaki S. & Marumo F. Phosphorylation of serine 256 is required for cAMP-dependent regulatory exocytosis of the aquaporin-2 water channel. J. Biol. Chem. 272, 14800–14804 (1997). 9169447 10.1074/jbc.272.23.14800

[b34] Nedvetsky P. I. *et al.* Regulation of aquaporin-2 trafficking. Handb. Exp. Pharmacol. 133–157 (2009). 19096775 10.1007/978-3-540-79885-9_6

[b35] Gilbard J. P., Rossi S. R., Heyda K. G. & Dartt D. A. Stimulation of tear secretion by topical agents that increase cyclic nucleotide levels. Invest. Ophthalmol. Vis. Sci. 31, 1381–1388 (1990). 2365569

[b36] Gilbard J. P., Rossi S. R., Heyda K. G. & Dartt D. A. Stimulation of tear secretion and treatment of dry-eye disease with 3-isobutyl-1-methylxanthine. Arch. Ophthalmol. 109, 672–676 (1991). 1709002 10.1001/archopht.1991.01080050086035

[b37] Klenkler B. & Sheardown H. Growth factors in the anterior segment: role in tissue maintenance, wound healing and ocular pathology. Exp. Eye Res. 79, 677–688 (2004). 15500826 10.1016/j.exer.2004.07.008

[b38] Dartt D. A. Neural regulation of lacrimal gland secretory processes: relevance in dry eye diseases. Prog. Retin. Eye Res. 28, 155–177 (2009). 19376264 10.1016/j.preteyeres.2009.04.003 PMC3652637

[b39] Gaal V. *et al.* Investigation of the effects of PACAP on the composition of tear and endolymph proteins. J. Mol. Neurosci. 36, 321–329 (2008). 18421426 10.1007/s12031-008-9067-5

[b40] Stern M. E. *et al.* The pathology of dry eye: the interaction between the ocular surface and lacrimal glands. Cornea 17, 584–589 (1998). 9820935 10.1097/00003226-199811000-00002

[b41] Takahashi Y. *et al.* Anatomy of secretory glands in the eyelid and conjunctiva: a photographic review. Ophthal. Plast. Reconstr. Surg. 29, 215–219 (2013). 10.1097/IOP.0b013e3182833dee 23381567

[b42] Fosgerau K. & Hoffmann T. Peptide therapeutics: current status and future directions. Drug Discov. Today 20, 122–128 (2015). 25450771 10.1016/j.drudis.2014.10.003

[b43] Nakamachi T. *et al.* IL-6 and PACAP receptor expression and localization after global brain ischemia in mice. J. Mol. Neurosci. 48, 518–525 (2012). 22669509 10.1007/s12031-012-9819-0

[b44] Suzuki R. *et al.* Expression of the receptor for pituitary adenylate cyclase-activating polypeptide (PAC1-R) in reactive astrocytes. Brain Res. Mol. Brain Res. 115, 10–20 (2003). 12824050 10.1016/s0169-328x(03)00172-4

[b45] Roach P. J., Laskin J. & Laskin A. Nanospray desorption electrospray ionization: an ambient method for liquid-extraction surface sampling in mass spectrometry. Analyst 135, 2233–2236 (2010). 20593081 10.1039/c0an00312c

[b46] Lanekoff I. *et al.* Automated platform for high-resolution tissue imaging using nanospray desorption electrospray ionization mass spectrometry. Anal. Chem. 84, 8351–8356 (2012). 22954319 10.1021/ac301909a

[b47] Lanekoff I. *et al.* High-speed tandem mass spectrometric in situ imaging by nanospray desorption electrospray ionization mass spectrometry. Anal. Chem. 85, 9596–9603 (2013). 24040919 10.1021/ac401760s PMC3867692

[b48] Ohtaki H. *et al.* Pituitary adenylate cyclase-activating polypeptide (PACAP) decreases ischemic neuronal cell death in association with IL-6. Proc. Natl Acad. Sci. USA 103, 7488–7493 (2006). 16651528 10.1073/pnas.0600375103 PMC1464366

