# Supplementary material for: PACAP suppresses dry eye signs by stimulating tear secretion
Source: Nat Commun. 2016 Jun 27;7:12034. doi: 10.1038/ncomms12034 (PMC4931240; doi:10.1038/ncomms12034)
Supplement: Supplementary Information — Supplementary Figures 1-14 and Supplementary Table 1 [file ncomms12034-s1.pdf]

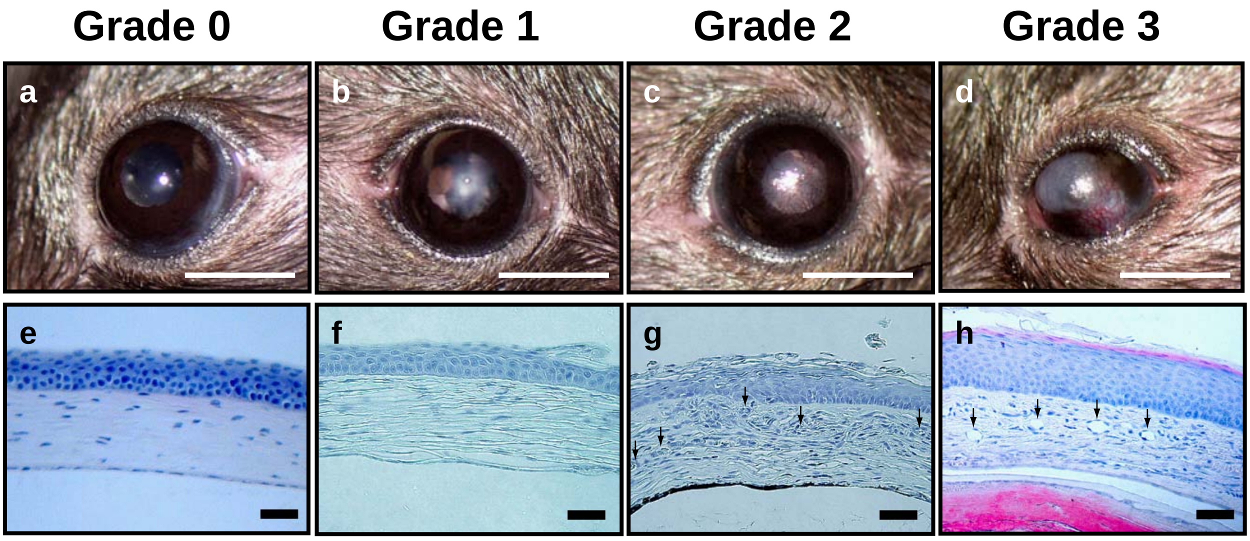

Supplementary Figure 1. Scale of corneal keratinization in mice. Low-power pictures of the corneal surface (**a-d**) and hematoxylin-eosin staining of the corneal surface (**e-f**) are shown. Keratinization of the cornea was classified into four grades (Grades 0-3) by visual observation as follows: (**a,e**) Grade 0 (normal) = no observable abnormality, (**b,f**) Grade 1 = clouded cornea caused by the irregularity of the substantia propia, (**c,g**) Grade 2 = angiogenesis in the substantia propia, and irregularities of the corneal surface, and (**d**,**h**) Grade 3 = hypertrophy of the corneal epithelium and keratinization. Scale bar, 2 mm in **a-d**, and 50 µm in **e-f**. Arrows indicate newly formed blood vessels.

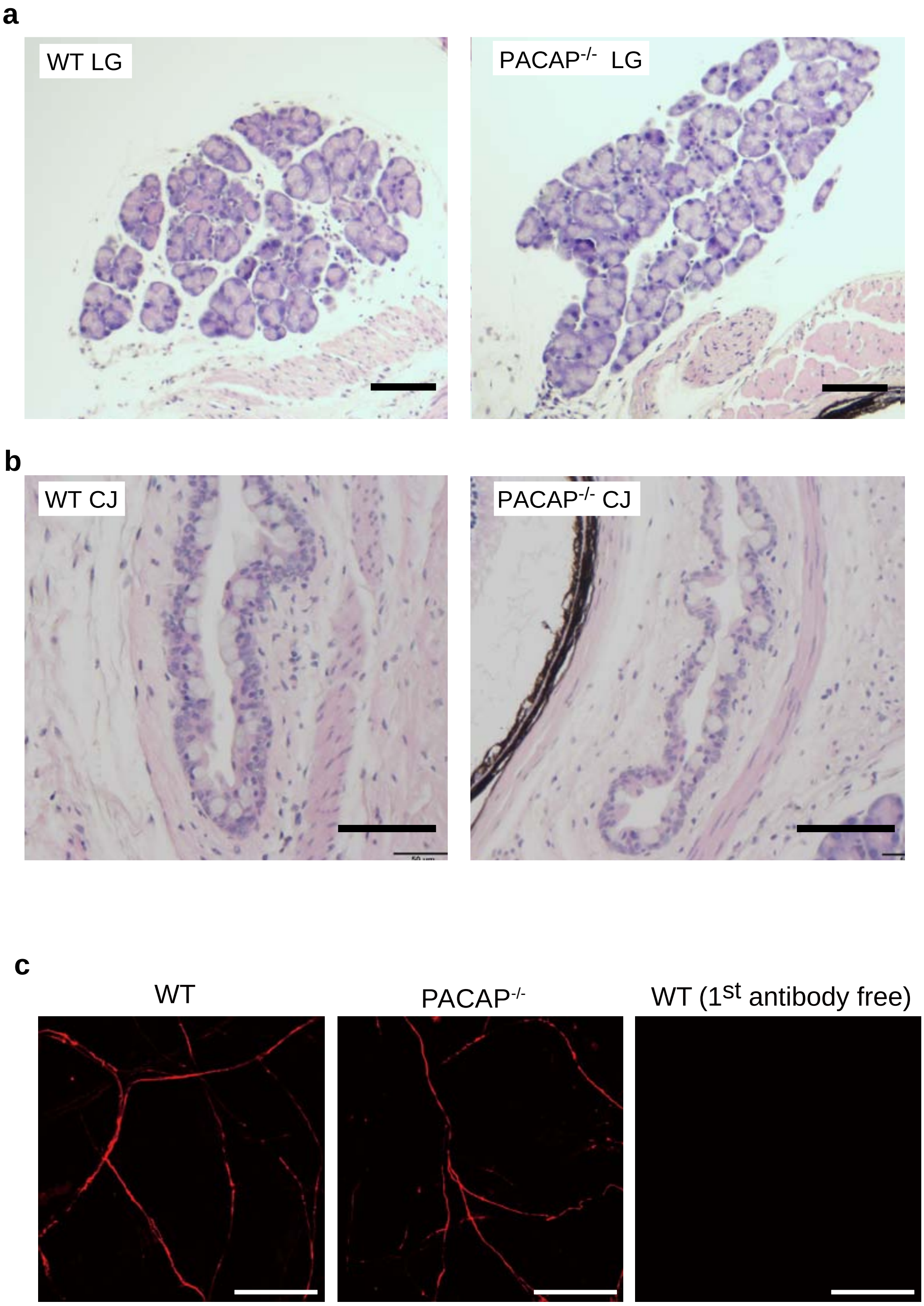

Supplementary Figure 2. Histological observation of lacrimal glands, conjunctival tissues and corneal nerve fibers in female wild-type and PACAP-/- mice. Representative pictures of the lacrimal glands (LG; **a**), conjunctival tissues (CJ; **b**) and Neurofilament 200 immunoreactivity on corneal flat mounts before the onset of keratinization (**c**) are shown. A wild-type cornea to which the primary antibody was not applied was used as a negative control. Scale bar, 50 µm in **a**, **b**, and 200 µm in **c**.

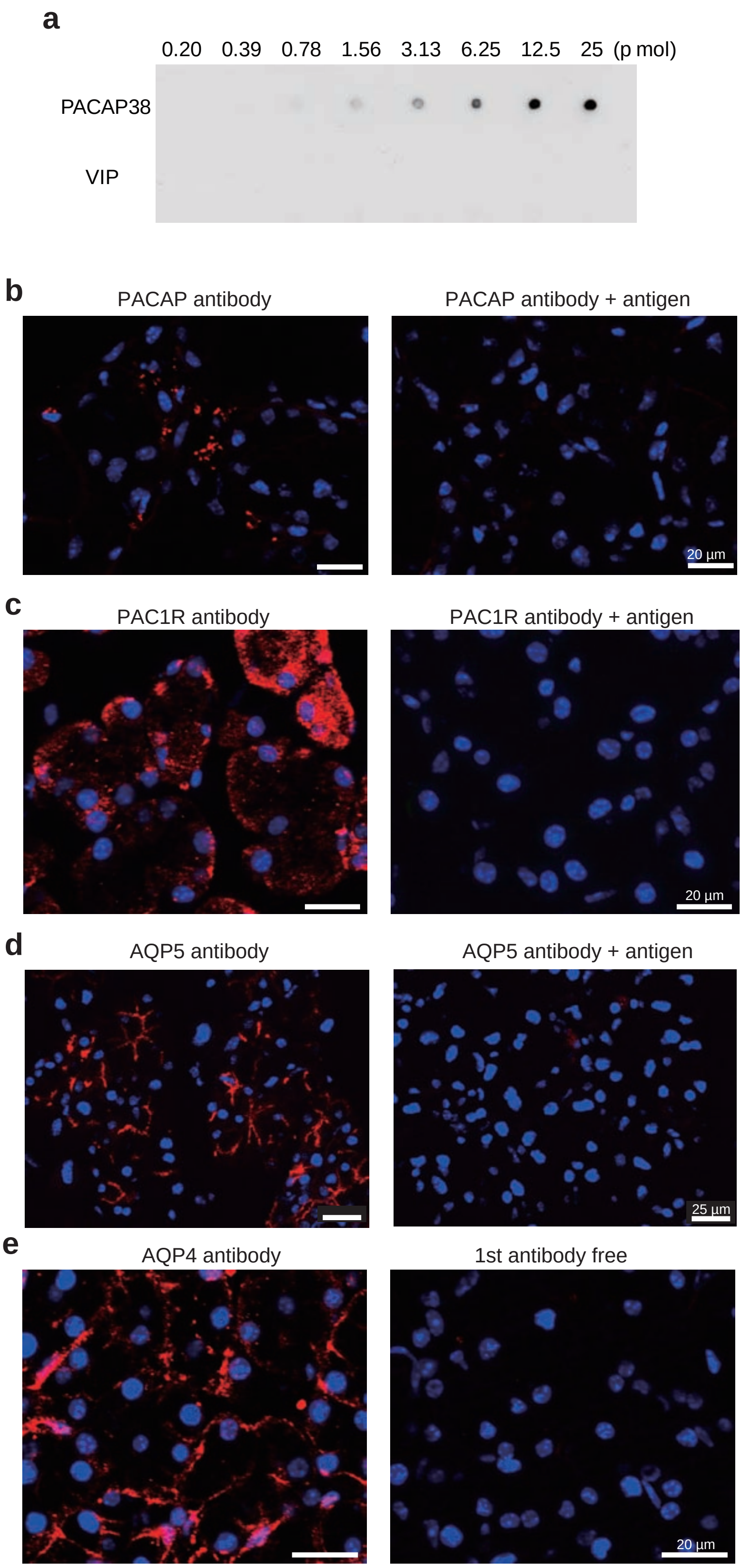

Supplementary Figure 3. Confirmation of the specificity of PACAP, PAC1-R, AQP5 and AQP4 antibodies. (**a**) Dot blotting analysis using PACAP antibody on a membrane treated with different doses of PACAP38 and VIP. (**b-d**) Antigen-absorption tests of PACAP, PAC1-R and AQP5 antibody on lacrimal gland section. (**e**) Comparison with AQP4 immunostaining and a primary antibody-free negative control. The same exposure times were used for the panels on the left and the right.

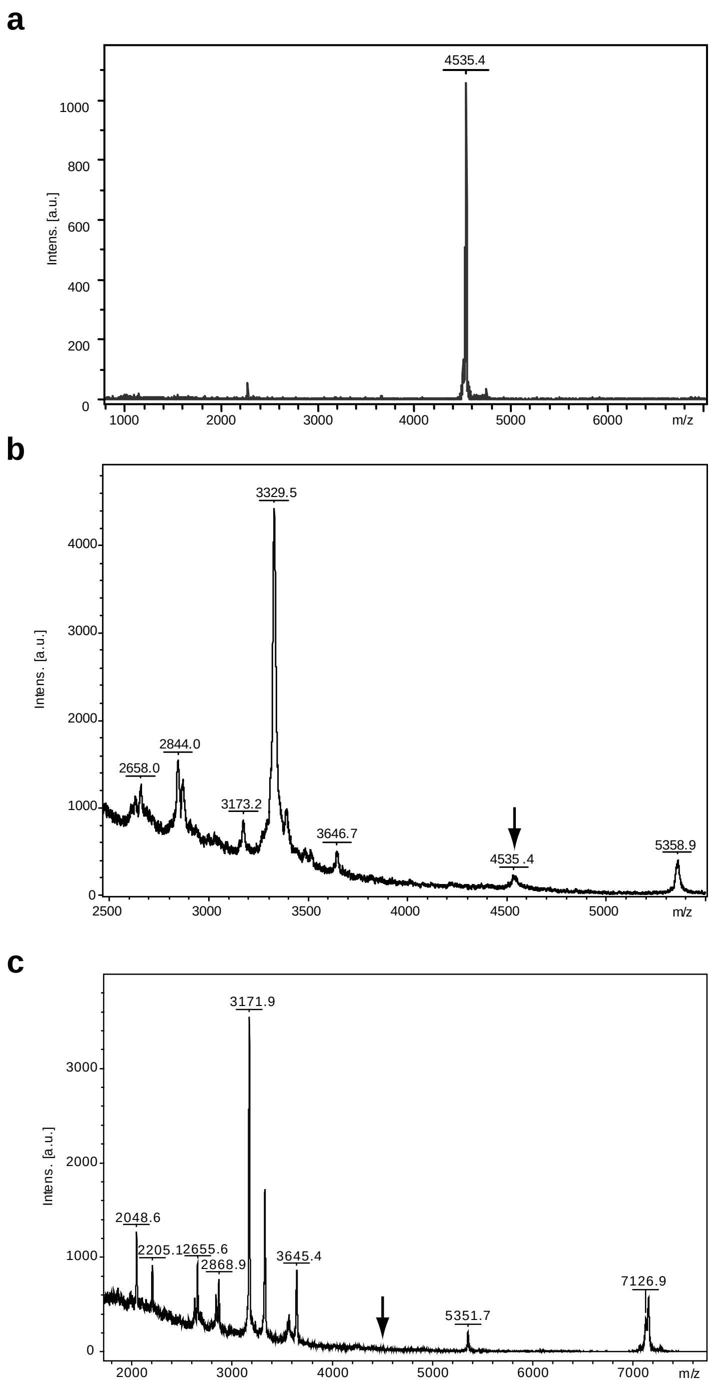

Supplementary Figure 4. MS spectrum analysis of mouse tears. (**a-c**) Typical MALDI TOF MS spectrum of the PACAP38 standard (**a**) and tears from wild-type (**b**) and PACAP-/- (**c**) mice. The position of the PACAP38 peptide peak (m/z 4535.4) is indicated by the black arrows in b and c.

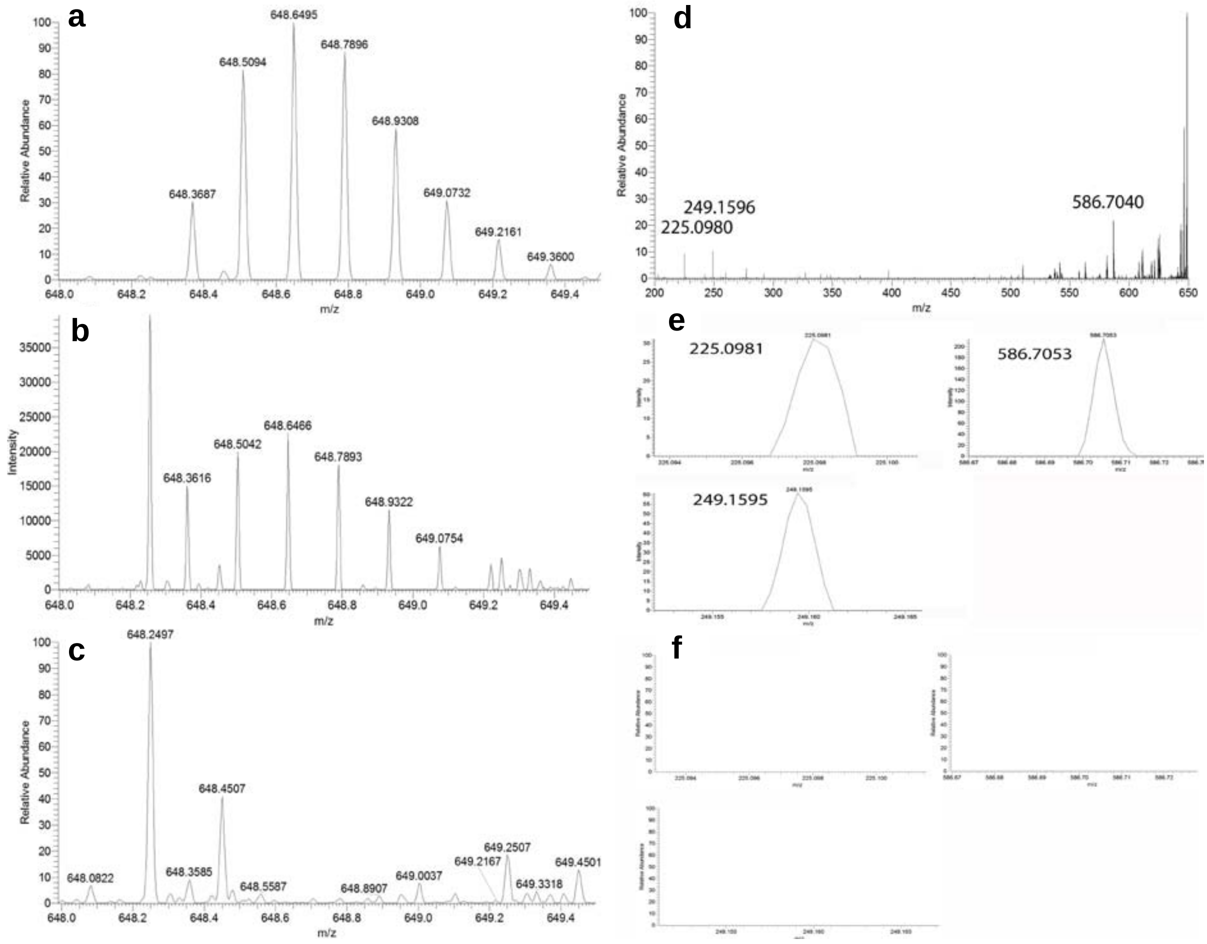

Supplementary Figure 5. Representative nano-DESI SIM spectra of 648.5 ±2 for (**a**) PACAP38 standard (z=7), (**b**) wild-type mouse sample, showing the peaks of PACAP38 and (**c**) PACAP-/- mouse sample without any peaks corresponding to PACAP38. The intensity of 100% relative abundance for the standard is 2.8e6 and 1.1e5 for the PACAP-/- mouse sample. Nano-DESI MS/MS spectra of 648.5 ±1 (precursor ion) for (**d**) PACAP38 standard with the m/z of the three most intense fragments annotated, (**e**) three mass spectra zoomed in to show the respective three fragments from the wild-type mouse sample and (**f**) three mass spectra showing the same m/z range as in **b** without any detected fragments for the PACAP-/- mouse sample.

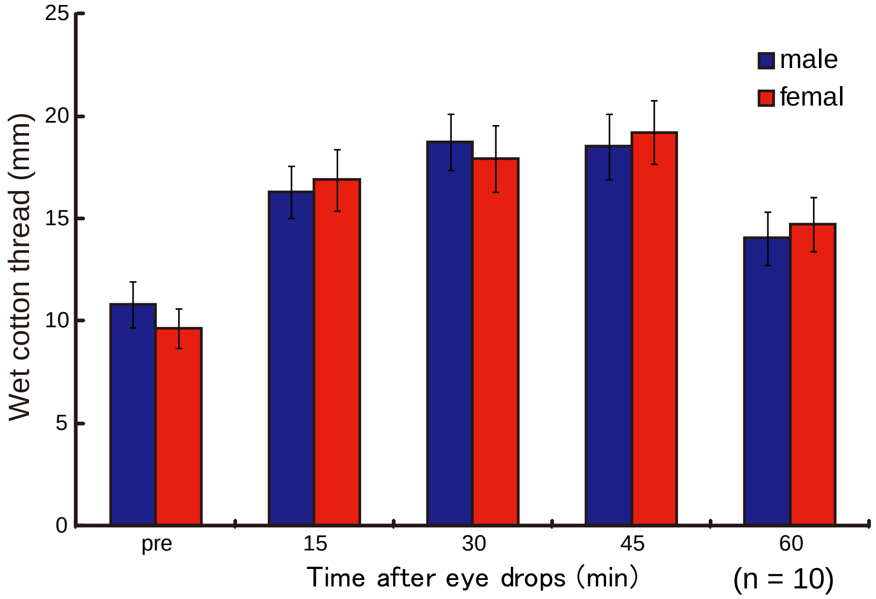

Supplementary Figure 6. PACAP eye drop test in male and female wild-type mice. PACAP (10^{-10} M) was administered in the form of eye drops, and the tear secretion level was measured using the cotton thread method. The basal tear secretion level and the PACAP-induced tear secretion level did not differ significantly between the male and female animals (one-way ANOVA).

a

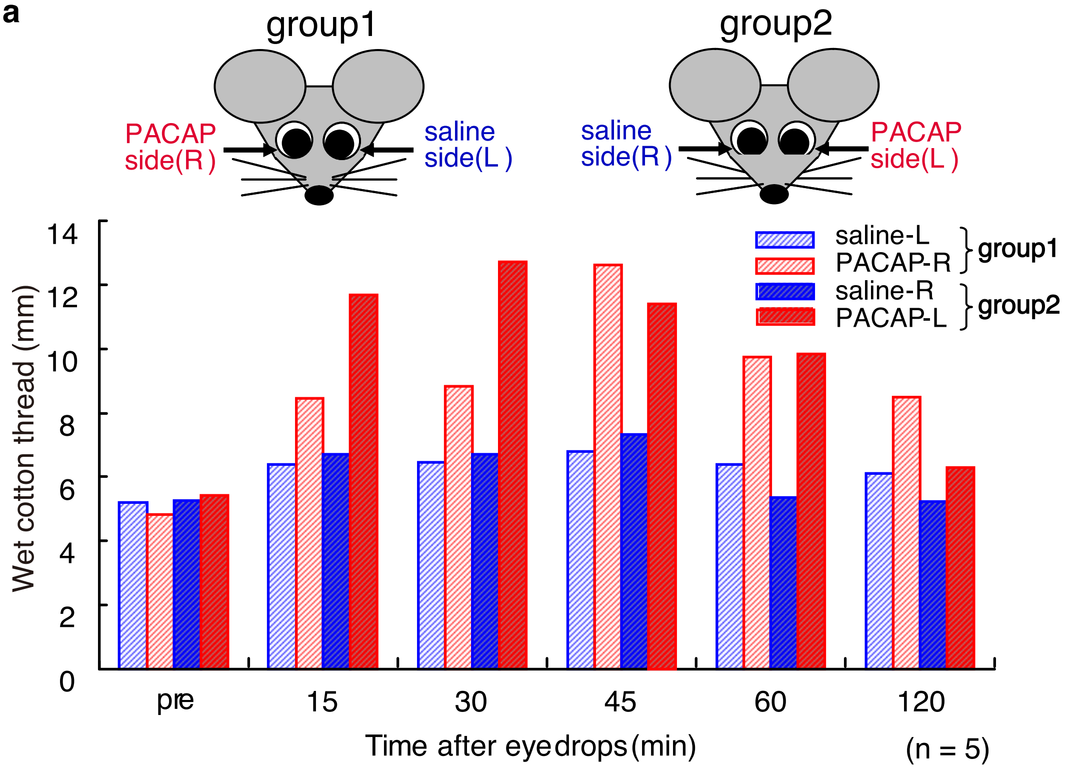

b

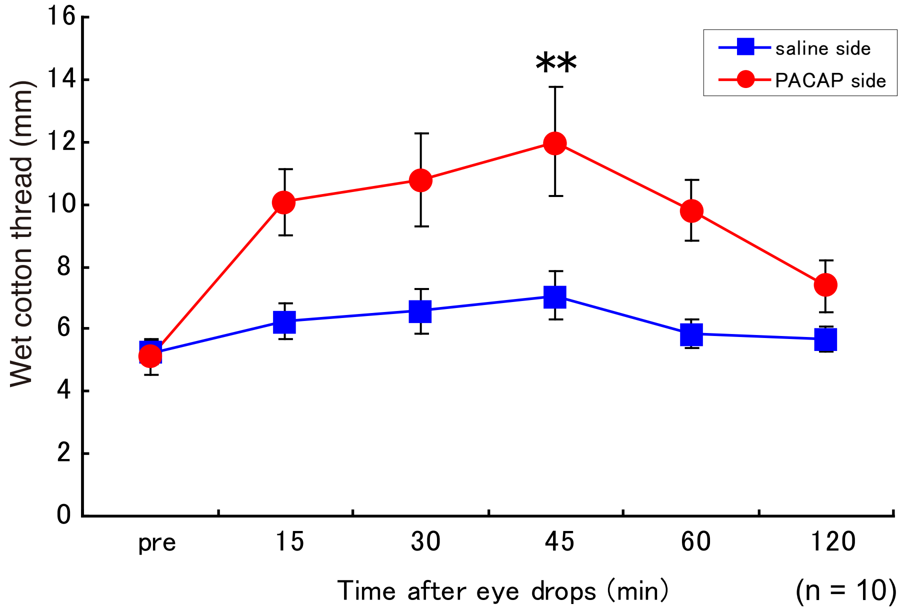

Supplementary Figure 7. Unilateral PACAP eye drop test in male wild-type mice. (**a**) Two groups were prepared. Group 1: PACAP38-treated right side and saline-treated left side; Group 2: saline-treated right side and PACAP38-treated left side (n = 5 per group). (**b**) Summary of both groups (n = 10 per side, one-way ANOVA). PACAP only induced tear secretion on the PACAP38-treated side.

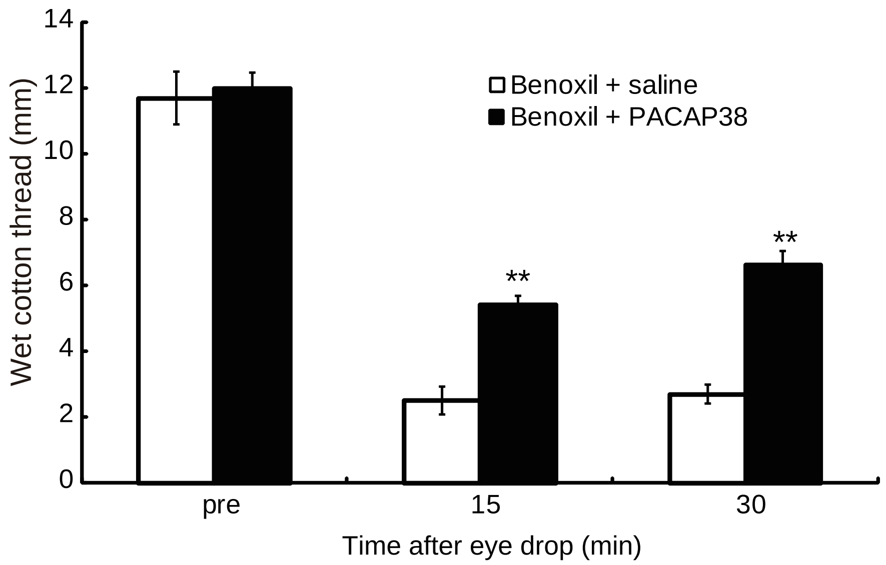

Supplementary Figure 8. Correlation between PACAP-induced tear secretion and corneal reflection. Following pre-treatment with the topical anesthetic, Benoxil, the tear secretion level with or without PACAP38 eye drops was examined in male wild-type mice (n = 10 per group, one-way ANOVA). **:$P < 0.01$ vs. the saline-treated group.

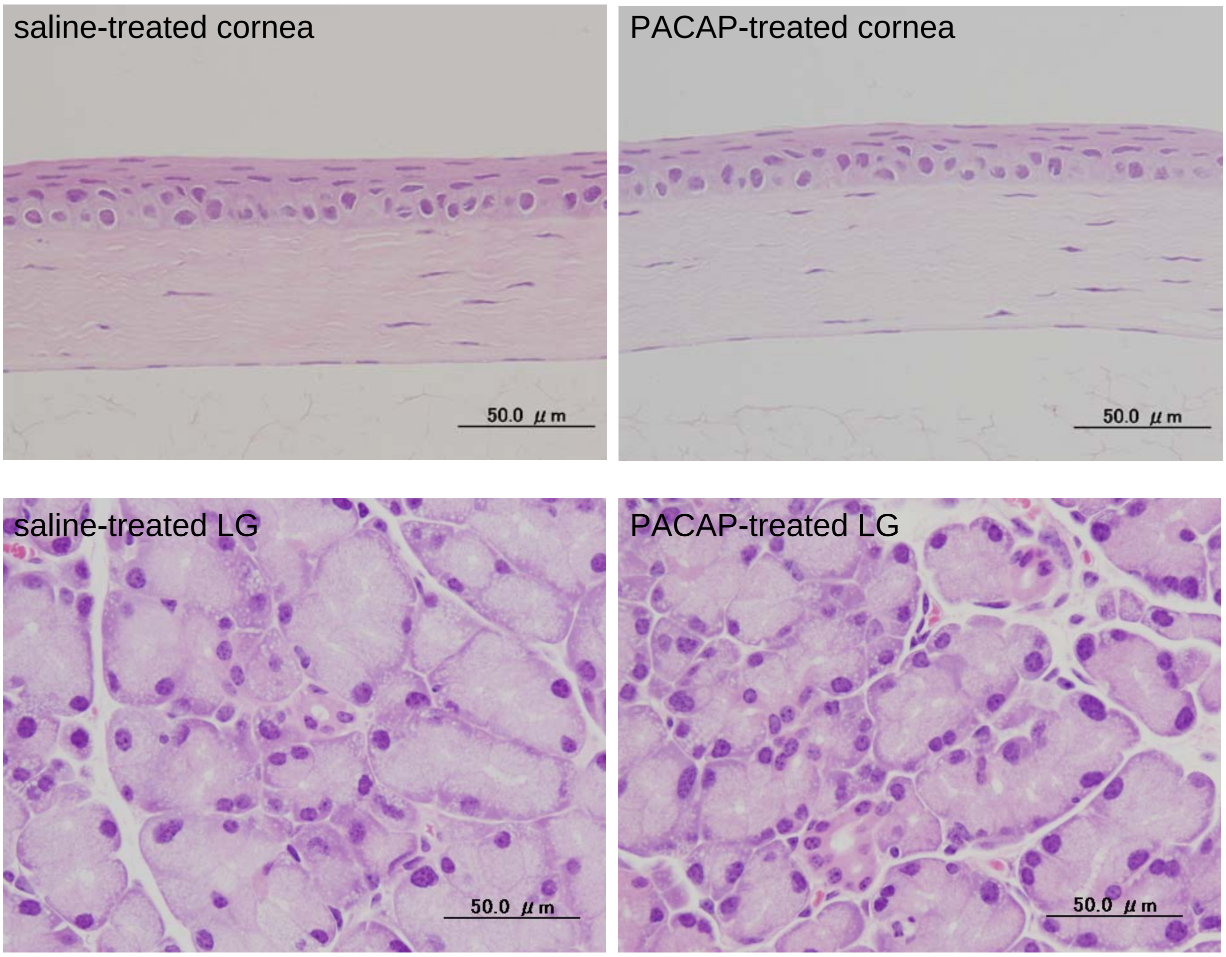

Supplementary Figure 9. Histopathological observation of the cornea and lacrimal gland after administration of PACAP eye drops. The cornea and lacrimal gland from a male wild-type mouse were examined 48 h after application of 10^{-7} M PACAP or saline eye drops. No pathological changes were observed.

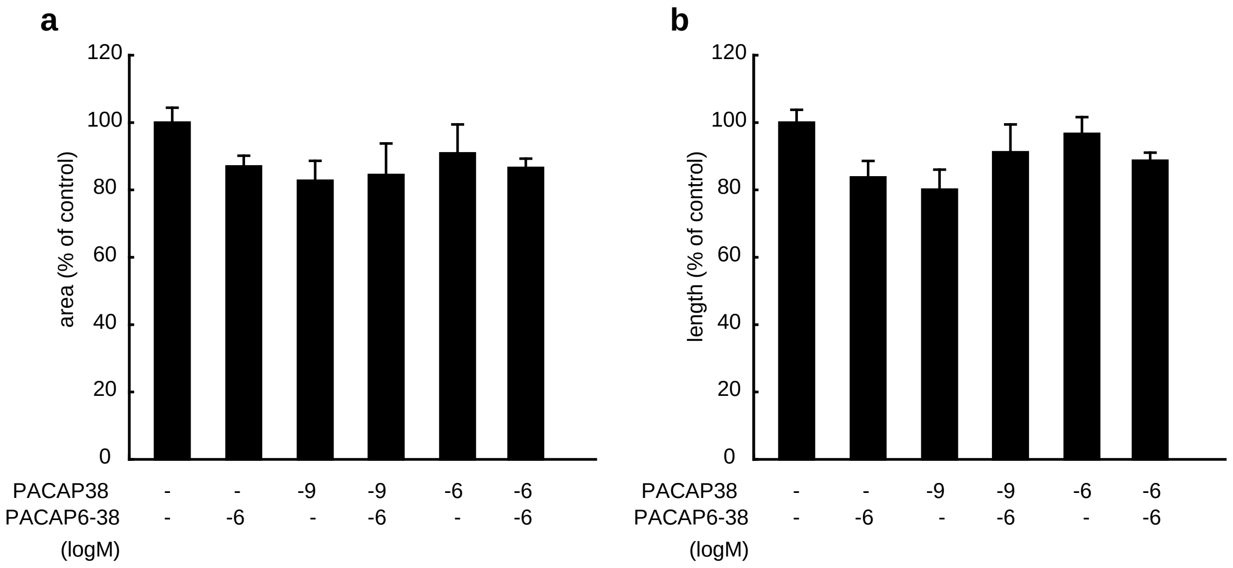

Supplementary Figure 10. Effect of PACAP on angiogenesis in vitro. Effect of PACAP and PACAP6-38 on tube formation by endothelial cells assessed with an angiogenesis kit using a human umbilical vein endothelial cell and fibroblast co-culture system. Administration of PACAP at 10^{-9} or 10^{-6} M with or without 10^{-6} M PACAP6-38 did not affect either the area of CD31-positive tubes (**a**) or the length of the CD31-positive tubes (**b**) in culture (n = 6 per group). There was no significant difference between the treatment groups (one-way ANOVA test)

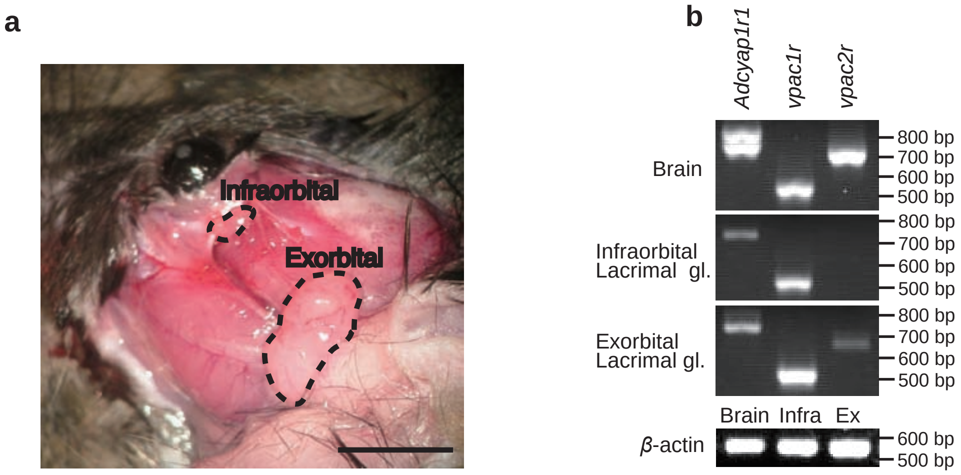

Supplementary Figure 11. Expression of PACAP receptors mRNA in infraorbital and/or exorbital lacrimal glands. (**a**) Macroscopic images of the infraorbital and exorbital lacrimal glands, in male mice. Scale bar, 5 mm. (**b**) PACAP receptor mRNA expression in the infraorbital and exorbital lacrimal glands detected by RT-PCR.

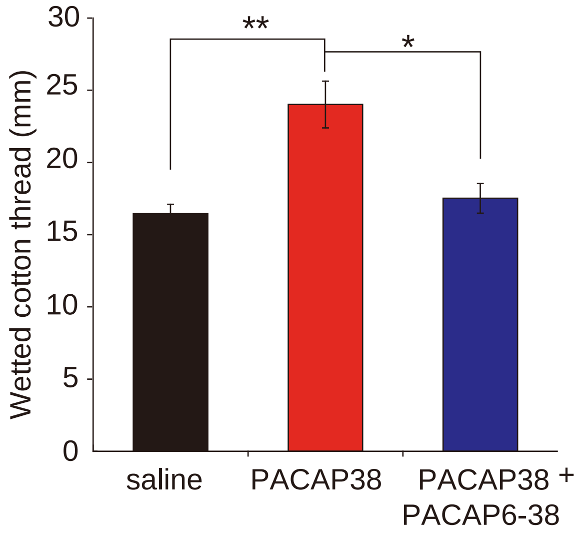

Supplementary Figure 12. Systemic infusion of PACAP38 in male wild-type mice. Effects of systemic injection of PACAP38 or PACAP38 with PACAP6-38 on tear secretion in mice (n = 6 per group, one-way ANOVA). *P < 0.05, **P < 0.01.

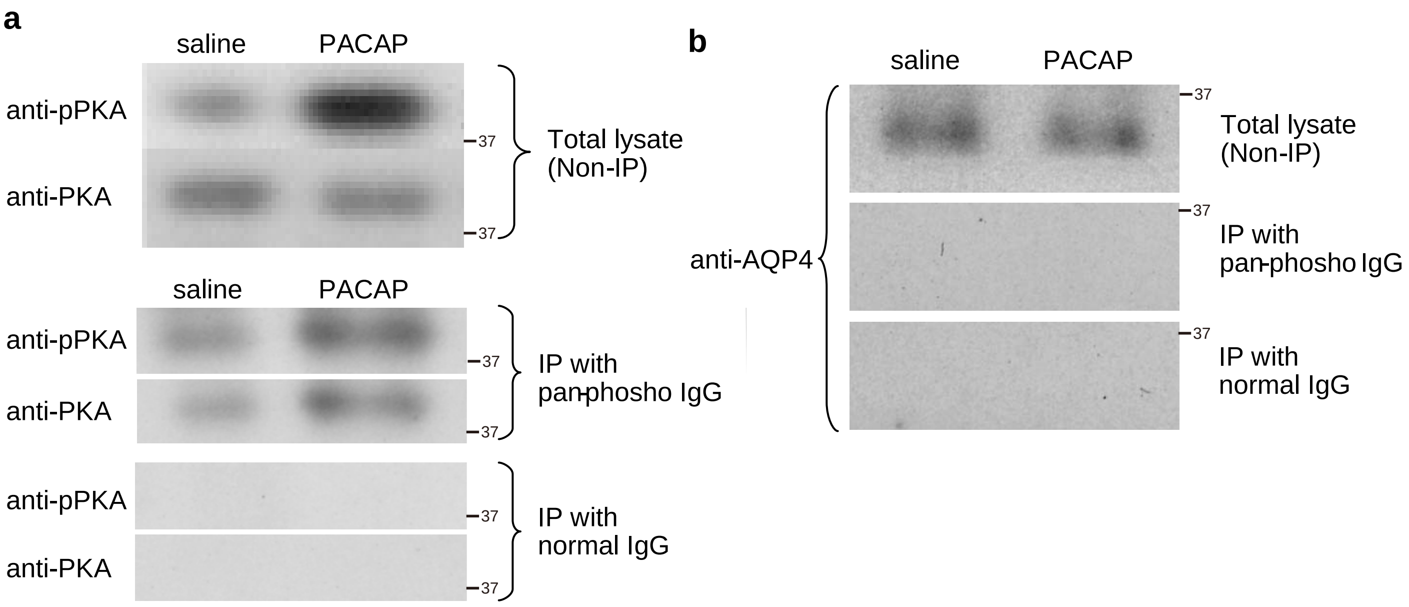

Supplementary Figure 13. Western blot analysis using saline- or PACAP-treated infraorbital lacrimal glands of male wild-type mice and an immunoprecipitation (IP) method. (**a**) Detection of PKA and pPKA with either an anti-PKA antibody with recognition for total PKA, including pPKA, or an anti-pPKA-specific antibody in the total lysate, or after IP with either a pan-phospho IgG or a normal IgG. The pPKA signal was increased in the total lysate from PACAP-treated lacrimal glands, but the total PKA signal did not appear to change. Both pPKA and total PKA signals were increased in the extracts from the PACAP-treated lacrimal glands that were immunoprecipitated with a pan-phospho IgG, but there was no detectable signal in any extract that was immunoprecipitated with a normal IgG. (**b**) Detection of AQP4 with an anti-AQP4 antibody in the total lysate, or after IP with either a pan-phospho IgG or a normal IgG. The AQP4 signals in the total lysate did not differ between saline- and PACAP-treated lacrimal glands. AQP4 signals were not detected after IP with either a pan-phospho IgG or a normal IgG.

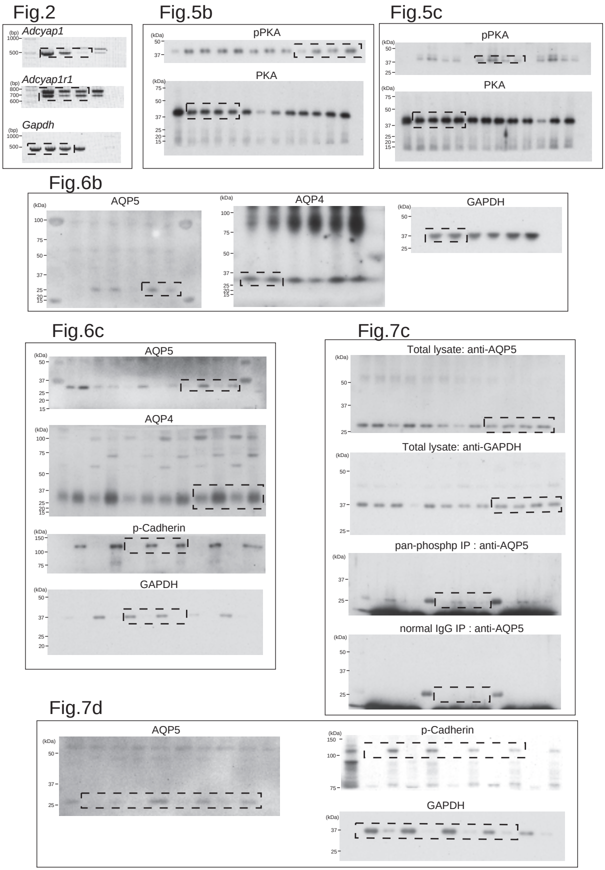

Supprementary Figure14. Uncropped images. Dashed boxes show cropped regions.

Supplementary Table 1. The sequences of mouse AQP5 and control siRNAs.

siRNA NAME	5'- Sequence -3'	Grade	MW
mAQP5#1	CCA UCG AG C UGA CGG CAC AdTdT	HPLC	6667.1
	UGU GCC GUC AGC UCG AUG GdTdT	HPLC	6678.1
mAQP5#2	GGA UGG GAU GGG AGC AGA AdTdT	HPLC	6891.3
	UUC UGC UCC CAU CCC AUC CdTdT	HPLC	6438.9
mAQP5#3	GCU CUU CAG GAG AGA GAU AdTdT	HPLC	6733.2
	UAU CUC UC U CCU GAA GAG CdTdT	HPLC	6567.0
Negative	UAC UAU UCG ACA CGC GAA GdTdT	HPLC	6653.1
control	CUU CGC GUG UCG AAU AGU AdTdT	HPLC	6647.1

Three types of mouse AQP5 siRNA and non-target negative control siRNAs were designed and purchased from BONAC Corporation.
